# MCOLN2 (Mucolipin-2) promotes autophagy to confer Adriamycin resistance in breast cancer by regulating mTOR degradation

**DOI:** 10.1007/s13402-026-01270-x

**Published:** 2026-08-03

**Authors:** Hongyan Yu, Jie Wang, Yongchen Ji, Xiaofang Liu, Mingxu Xie, Chun Yin Lo, Wenhan Cao, Huazhong Zhou, Xiaoxue Li, Xiaoling Huang, Liwen Jiang, Xiaoqiong Gu, Xiangqi Meng, Xiaoqiang Yao

**Affiliations:** 1 Department of Clinical Biological Resource Bank, Guangzhou Women and Children’s Medical Center, Guangzhou Medical University, Guangzhou, China; 2 School of Biomedical Sciences and Li Ka Shing Institute of Health Science, Faculty of Medicine, The Chinese University of Hong Kong, Hong Kong, China; 3 Guangdong Provincial Key Laboratory of Colorectal and Pelvic Floor Diseases, Guangdong Institute of Gastroenterology, The Sixth Affiliated Hospital, Sun Yat-sen University, Guangzhou, China; 4 Biomedical Innovation Center, The Sixth Affiliated Hospital, Sun Yat-sen University, Guangzhou, China; 5 Department of Radiation Oncology, Dalian Municipal Central Hospital, Dalian, China; 6 Department of Blood Transfusion, The Third Affiliated Hospital of Sun Yat- sen University, Guangzhou, China; 7 National Key Laboratory for Development and Utilization of Forest Food Resources, Zhejiang A&F University, Hangzhou, China; 8 Centre for Cell & Developmental Biology, School of Life Sciences, The Chinese University of Hong Kong, Hong Kong, China; 9 College of Veterinary Medicine, South China Agricultural University, Guangzhou, China

**Keywords:** Breast cancer, MCOLN2, Autophagy, Adriamycin resistance, mTOR

## Abstract

**Background:**

Adriamycin is a cornerstone chemotherapeutic agent for advanced breast cancer, but its efficacy is frequently compromised by chemoresistance, a process in which macroautophagy is critically involved. However, the underlying mechanisms driving this autophagy-mediated resistance remain poorly understood.

**Methods:**

Clinical data from patients with breast cancer who underwent chemotherapy were collected and the expression of MCOLN2 in tumor tissues was assessed. Using Adriamycin-resistant MCF-7/ADM cells and their parental MCF-7 cells, we investigated the role of MCOLN2 in regulating autophagic flux and mediating drug resistance. Key findings were further validated using an in vivo xenograft model.

**Results:**

MCOLN2 expression was significantly upregulated in Adriamycin-resistant MCF-7/ADM cells and chemoresistant patient samples, where high levels correlated with poor prognosis. Knockdown of MCOLN2 sensitized MCF-7/ADM cells to Adriamycin both in vitro and in vivo, whereas its overexpression conferred resistance in parental MCF-7 cells. Mechanistically, MCOLN2 promoted autophagy, as evidenced by increased LC3-II levels and autophagosome formation. This pro-autophagic effect was mediated by MCOLN2-induced destabilization and lysosomal degradation of mTOR. Consequently, pharmacological or genetic inhibition of autophagy effectively reversed the MCOLN2-driven chemoresistance.

**Conclusion:**

The MCOLN2-mTOR-autophagy axis has a critical role in the development of therapeutic resistance to Adriamycin in breast cancer. Targeting MCOLN2 could be a promising therapeutic strategy to overcome Adriamycin resistance in breast cancer patients.

**Supplementary Information:**

The online version contains supplementary material available at https://doi.org/10.1007/s13402-026-01270-x.

## Introduction

Breast cancer is the most commonly diagnosed cancer and the leading cause of cancer death in women worldwide [1]. Despite recent progress in the field, clinical challenges including chemoresistance, recurrence, and metastasis remain major obstacles. Chemotherapy is the standard treatment for patients with triple-negative, HER2-positive, and advanced luminal breast cancer. Unfortunately, tumors eventually develop chemoresistance, which reduces the efficacy of chemotherapeutic agents [2]. Therefore, exploring the novel mechanisms underlying chemoresistance in breast cancer is of pivotal significance.

Cancer cells adopt multiple strategies to acquire chemoresistance, including overproduction of efflux transporters that can remove chemotherapeutic drugs from tumor cells, the presence of cancer stem cells that are inherently resistant to chemotherapeutic drugs, and the establishment of a reversed pH gradient across the cancer cell membrane (pHe < pHi) to interfere with drug entry [3]. Autophagy, also known as macroautophagy, is another mechanism that plays a key role in cancer cell chemoresistance. Autophagy is regulated by a set of autophagy-related genes (ATGs). During the autophagic process, the cytosolic form of microtubule-associated protein 1 light chain 3 (LC3-I) is conjugated to phosphatidylethanolamine [4] to form the membrane-bound form LC3-II, which initiates the formation and elongation of autophagosomes. mTOR (mammalian target of rapamycin) is a pivotal inhibitor of autophagy [5]. At the autophagy induction phase, mTORC1 suppresses the activity of the ULK1 complex by phosphorylating its key components (ULK1 and ATG13), thereby inhibiting the initiation of autophagy [6]. Autophagy plays a dual role in regulating chemoresistance. In most cases, the autophagic process helps cancer cells survive under chemotherapy, leading to chemoresistance [7]. However, excessive autophagy can induce cancer cell death [3, 7].

Ca²⁺ is an important second messenger that participates in the regulation of chemoresistance [8, 9]. Most previous studies have focused on the role of plasma membrane Ca²⁺-permeable channels (e.g., transient receptor potential (TRP) subfamilies TRPC, TRPM, and TRPV) in chemoresistance [8, 9]. Unlike other TRP channels, members of the mucolipin/TRPML subfamily are Ca²⁺/Zn²⁺-permeable channels exclusively localized to the endolysosomal membrane [10]. Functionally, mucolipins/TRPMLs play a pivotal role in autophagy and lysosomal exocytosis in multiple cell types, including cancer cells [11, 12]. The mucolipin/TRPML subfamily consists of three members, namely mucolipin-1, -2, and − 3 (MCOLN1, -2, and − 3). While numerous studies have investigated MCOLN1 in cancer cells [13], only a few studies have explored the role of MCOLN2 in cancer. For instance, Morelli and Santini demonstrated that MCOLN2 promotes glioma tumor growth, which is associated with poor prognosis [14, 15]. Recently, our group reported that MCOLN2 facilitates prostate cancer progression via the IL-1β/NF-κB pathway [16]. However, to date, no studies have explored the role of MCOLN2 in breast cancer and its associated chemoresistance.

In the present study, we hypothesize that MCOLN2 may promote the autophagic process, thereby enhancing chemoresistance in breast cancer cells. A variety of methodologies, including Adriamycin-sensitive cancer cell growth assays, LC3-II-based autophagic flux analysis, mTOR activity and stability assessment, and tumor xenograft growth in immunodeficient NCG mice, were used to test this hypothesis. The results establish a critical role for MCOLN2 in the regulation of the mTOR-autophagy signaling axis within Adriamycin-resistant breast cancer cells. Our findings identify MCOLN2 as a compelling therapeutic target for reversing Adriamycin resistance and enhancing patient prognosis.

## Materials and methods

### Patient samples

Breast cancer specimens (excision/mastectomy specimens after chemotherapy) along with the corresponding clinical pathologic data from 44 patients were collected as described before [17]. All patients have received clinical circles of anthracycline-based chemotherapy. Tumor assessment was performed by haematoxylin and eosin (H&E) staining as a commonly used protocol for tumor diagnosis. The histological response to chemotherapy was evaluated by Miller & Payne grading system, which is a five-point grading system based on the reduction level of tumor cellularity. Patients with grade 1 and 2 had tumor area reduced by < 30% after chemotherapy, thus were considered non-responders of chemotherapy. Patients with grade 4 and 5 had tumor area reduced by more than 90%, and thus were considered responders of chemotherapy [18].

### Human tissue microarray and scoring

Immunohistochemistry staining for MCOLN2 was performed on human breast cancer tissue microarray specimens (HBreD140Su06, Xinchao, China), using the MCOLN2 antibody (1:400, Alomone labs). The original immunohistochemistry slides were scanned by Slide Scanning System SQS-1000 (TEK-SQRAY). The percentage of MCOLN2-positive cancer cells and the staining intensity grade (0–3) were determined. The staining intensity and percentage were analyzed by Halo pathology software (Indica Labs).

### Bioinformatics analysis

The clinical significance of MCOLN2 was evaluated through bioinformatics analyses. Differential expression of MCOLN2 between breast cancer and normal tissues was assessed using data from the Gene Expression Omnibus (GEO) database (https://www.ncbi.nlm.nih.gov/geo/). For survival analysis, patients were dichotomized into high- and low-MCOLN2 expression groups based on the median expression value, and overall survival (OS) was estimated using the Kaplan–Meier method. Differences in survival between the two groups were compared using the log-rank test, with statistical significance set at *P < 0.05.

### Cell lines and culture

MCF-7 cells were from the American Type Culture Collection. The Adriamycin-resistant MCF-7/ADM cells were established by treating the cells with stepwise increasing concentrations of Adriamycin [8]. Adriamycin-resistant MDA-MB-231/ADM cells were brought from Anwei biology company (#AW-CH0853). Cells were cultured in DMEM supplemented with 10% FBS, 100 µg/ml streptomycin and 100 U/ml penicillin.

### Colony formation assays

Briefly, single-cell suspension was plated at a density of 500 cells per well in 6 well plates. The culture medium (contain Adriamycin) was replaced every three days. Two weeks later, the formed clones were fixed and stained with 0.5% crystal violet, and the colonies with more than fifty cells were counted under a microscope.

### Cell viability assay

Cell growth was determined by cell counting kit-8 (CCK-8) (#c0038, Beyotime). Cells at a density of 2 × 10^3^ per well were grown in the 96 well plates in 0.1 ml full medium at 37 °C for different periods of time. Each treatment had five duplicated wells, and CCK-8 assays were repeated three times according to the manufacturer’s protocol.

### RNA extraction and RT-PCR

Total RNA was extracted using TRIzol (Invitrogen). The cDNA was synthesized from 2 µg of total RNA using M-MLV reverse transcriptase (Thermo Fisher Scientific) for quantitative RT-PCR. The primer sequence of target genes and reference gene GAPDH were listed in Supplemental Table 1.

### Immunocytochemistry

The cells grown on coverslips were washed with PBS twice, fixed in 4% paraformaldehyde (Sigma-Aldrich, USA) in PBS, permeabilized with 0.1% Triton X-100 (Sigma-Aldrich, USA), and then blocked with 5% bovine serum albumin (Thermo Fisher Scientific) in PBS for 1 h at room temperature. The cells were incubated with primary antibodies at 4 °C overnight, followed by incubation with fluorescence-conjugated secondary antibodies for 90 min at room temperature in the dark. Images were acquired using a Confocal Laser Scanning Imaging System (Fluoview 1200; Olympus, Tokyo, Japan).

### Protein turnover assay

Protein turnover was performed as previously described [19]. Briefly, cells were treated with 100 µg mL^− 1^ cycloheximide and harvested at indicated time points after cycloheximide treatment. The protein levels were analyzed by immunoblots.

### LC3 puncta quantification

Cells were incubated with GFP-LC3B tandem reporter adenovirus (C3006, beyotime) for 24 h. Following the indicated treatments, fluorescent images were acquired using a Confocal Laser Scanning Imaging System (Fluoview 1200; Olympus, Tokyo, Japan). GFP-LC3 puncta per cell were analyzed using ImageJ software.

Similar measurement was used for mCherry-GFP-LC3 puncta quantification. Briefly, cells transfected with either siNC or siMCOLN2 were further infected with the mCherry-GFP-LC3 tandem reporter adenovirus (C3011, beyotime) for 24 h. Following the indicated treatments, cells were fixed and images captured. Yellow and red puncta were counted for each cell. The number of yellow puncta represented autophagosome while red puncta represented autolysosomes.

### Lentivirus production and Doxycycline inducible cells construction

HEK293FT cells were seeded in 9-cm dishes. The cells with 60–80% confluence was co-transfected with pLKO-Tet-On (Novartis), 7.5 µg psPAX2 (Addgene, 12260) and 7.5 µg pMD2.G (Addgene, 12259) using a commercial lentivirus package kit (Biowit, Shenzhen, China) according to manufacturer’s instruction. The supernatants were harvested at 48 and 72 h, centrifuged at 400 g for 10 min, and then passed through 0.45-µm filters. The collected virus was stored at -80 °C. The shRNA sequences of MCOLN2 were listed in Supplementary Table 2.

Breast cancer cells were seeded in 6 well plates. When the cells reached 40% confluence, lentiviral conditional medium was added. After 48 h, the cells were treated with 2 µg/ml puromycin (Thermo Fisher Scientific) for 4 days to select positively infected cells. And the MCOLN2 shRNA expression was induced by 100 ng/ml doxycycline (Sigma-Aldrich) for 48 h. MCOLN2 knockdown was confirmed with qRT-PCR.

### Immunoblots

Total proteins were extracted from cells using cell lysis buffer containing PMSF (Beyotime). The samples were separated by 8–12% SDS-polyacrylamide gel electrophoresis, followed by transferring to PVDF membrane. After blocking in PBS containing 5% bovine serum albumin, the membrane was incubated with primary antibody at 4 °C overnight, followed by incubation with a peroxidase-linked secondary antibody (Thermo Fisher Scientific) at room temperature for 1 h. The signals were detected by X-ray film after incubating with immunoblotting Luminol reagent (GE Health care). Primary antibodies: MCOLN1(1:1000, #ACC081, Alomone labs), MCOLN2 (1:1000, #ACC082, Alomone labs), MCOLN3(1:1000, #ACC083, Alomone labs), GAPDH (1:50000, #60004, Proteintech), β-actin (1:5000, #AF7018, Affinity), LC3I/II(1:1000, #NB100-2220, Novus), Flag-tag (1:4000, #F1804, Sigma), Phospho-mTOR(Ser2448)(1:1000, #5536, CST), Phospho-4EBP1(Thr37/46)(1:1000, #2855, CST), Phospho-p70S6 Kinase (Ser389) (1:1000, #9234, CST), Phospho-ULK1 (Ser757) (1:1000, #6888T, CST), mTOR (7C10) (1:1000, #2983, CST), Myc-Tag (1:4000, #2276s, CST), p62 (1:1000, #5114, CST).

### siRNA transfection

Exponentially growing MCF-7/ADM cells were plated in 6 well plates for 24 h before transfection. When the cells were grown to 60–70% confluence, 30 pmol of siRNAs targeting the mRNAs of MCOLN2, mTOR or scrambled siRNA as negative control (siNC) were added with Lipofectamine RNAiMax reagent (ThermoFisher Scientific, #13778150). After treatment for at least 48 h, the interference efficiency was confirmed by qRT-PCR and immunoblots, and the cells were harvested for further assays. The siRNA oligonucleotides were synthesized from GenePharma (Shanghai, China) and the detailed sequences of the siRNA used in this study were listed in Supplementary Table 3.

### Transmission electron microscope

Ultrastructural analysis procedures for transmission electron microscope sample preparation, thin sectioning, and immunogold labeling of high-pressure frozen, freeze-substituted tissues and HM20 embedding were performed as described previously [20]. Briefly, MCOLN2 knockdown cells and the control cells (siNC) subjected to the indicated treatments were collected and immediately frozen in a high-pressure freezer (EM PACT2, Leica), followed by subsequent freeze substitution in dry acetone containing 0.1% uranyl acetate at -85 °C in an AFS freeze-substitution unit (Leica). Infiltration with Lowicryl HM20, embedding, and UV light polymerization were performed stepwise from − 85 °C to -10 °C. Sections were examined using a H-7650 transmission electron microscope with a charge-coupled device camera operating at 80 kV (Hitachi High-Technologies; www.hitachi-hightech.com/jp/). Autophagosomes were counted in at least 6 randomly selected intact cells across multiple independent fields per group. The average number of autophagosomes per cell was calculated for statistical analysis.

### Flow cytometry analysis

Flow cytometry was used to measure intracellular Adriamycin accumulation and apoptosis. MCF-7/ADM cells were transfected with siNC or siMCOLN2. After 48 h, the cells were treated with 4 µM Adriamycin for 24 h. For fluorescence analysis, cells were trypsinized, washed twice with ice-cold PBS, and resuspended in PBS. Adriamycin intrinsic fluorescence was immediately acquired through the PE channel. For apoptosis analysis, cells were washed once with ice-cold PBS and resuspended in Annexin V binding buffer. The cells were then stained with Annexin V-FITC and propidium iodide. Data were analyzed using FlowJo software (version 10.8.1).

### In vivo xenograft assay

Twenty-four NCG mice (five to six week-old female) were used in this study.

1 × 10^7^ transfected MCF-7/ADM cells/100 ul of PBS per mouse were injected subcutaneously in the lower back of the NCG mice. Tumor length and width were measured every 3 days until the end of the experiment. Three weeks later, the mice were sacrificed by CO_2_ inhalation, and tumors were isolated and measured. Tumor volumes were calculated: Volume = Length × Width^2^ /2. Doxycycline (30 ug/gram of body weight) was administered by intraperitoneal injection every two days to induce the knockdown of MCOLN2 expression and PBS was as vehicle control. Adriamycin (2 mg/kg) was injected at the tumor sites every four days.

### Statistical analysis

All the data were presented as means ± SD (at least three biological replicates or three independent experiments). Statistical analysis was performed using GraphPad Prism (GraphPad software, San Diego, California, USA). Survival analysis was carried out using the Kaplan–Meier method, and the log-rank test was used to compare the survival curves. Student’s t-test or one-way ANOVA was used for comparison between groups. *P* ≤ 0.05 was considered statistically significant.

## Results

### Upregulation of MCOLN2 enhances Adriamycin resistance in breast cancer cells

**Fig. 1:**
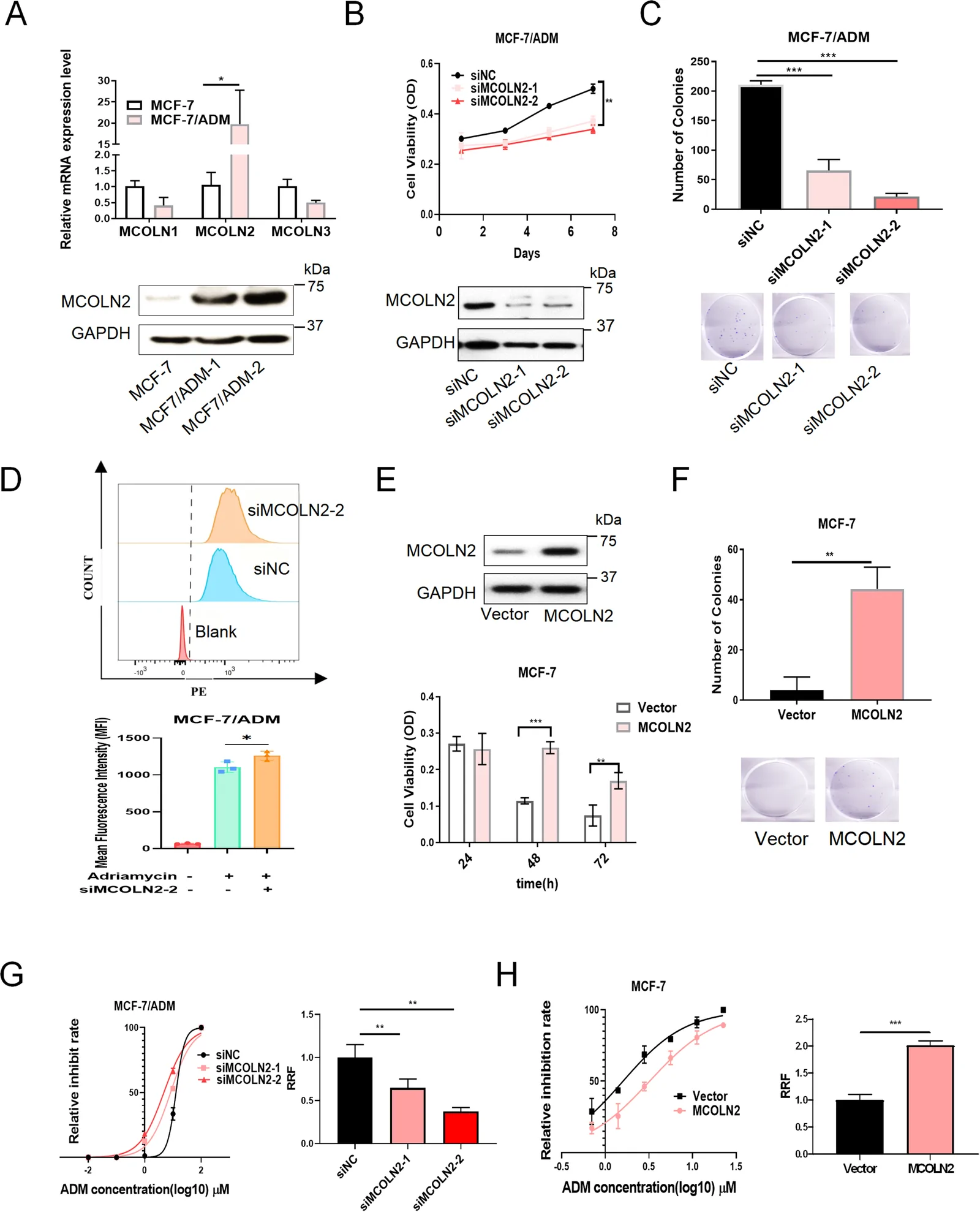
MCOLN2 upregulation enhances the Adriamycin resistance in breast cancer cells. (**A**) mRNA expression levels of the MCOLN family members were detected by qRT-PCR analysis (upper panel). The endogenous expression levels of MCOLN2 proteins in Adriamycin resistant (MCF-7/ADM) and their parental cells (MCF-7) were detected by immunoblots (lower panel). (**B**-**C**) The MCF-7/ADM cells were transfected with MCOLN2 siRNAs or negative control siRNAs (siNC). 48 h post transfection, the cells were incubated with 2 µM Adriamycin for different time durations in B or C. The cell viability was monitored by CCK8 assay (B, upper panel) and colony formation assay (C, data summary in upper panel and representative images in lower panel). The effectiveness of MCOLN2 siRNAs in knocking-down MCOLN2 expression was shown by immunoblots with GAPDH as loading control (B, lower panel). (**D**) Flow cytometry histograms showing Adrimycin fluorescence intensity distribution in MCF-7/ADM cells transfected with siMCOLN2-2, siNC. (**E**-**F**) Parental MCF-7 cells were transfected with MCOLN2 or empty vector plasmid. The effectiveness of MCOLN2 overexpression in elevating MCOLN2 protein level was shown by immunoblots with GAPDH as loading control (E, upper panel). 48 h post transfection, the cells were incubated with 1µM Adriamycin for indicated times. The cell viability was monitored by CCK8 assays (**E**) and colony formation assays (**F**). (**G**-**H**) Dose-dependent cell viability curves in response to different concentration of Adriamycin in MCF-7/ADM (**G**) and MCF-7 cells (**H**). Right: averages of relative resistant factors (RRFs, the ratio of IC50 values of experimental groups relative to that of control groups). Mean ± S.D. (*n* = 3). *, *P* < 0.05; **, *P* < 0.01; ***, *P* < 0.001

Adriamycin-resistant MCF-7 breast cancer cell lines (MCF-7/ADM) were established as reported previously [8]. Quantitative RT-PCR was performed to compare the mRNA levels of MCOLN family members between MCF-7/ADM cells and the parental MCF-7 cells. The results showed a ~ 20-fold increase of MCOLN2 mRNA expression in MCF-7/ADM cells compared with that in MCF-7 cells, while no obvious difference was observed in the expression of MCOLN1 and MCOLN3 between these two cell lines (Fig. 1A, upper panel). Immunoblots data confirmed the higher expression of MCOLN2 in MCF-7/ADM than in MCF-7 while MCOLN1 and MCOLN3 proteins remain unchanged. (Fig. 1A, supplemental Fig. 1A-B).

To determine the role of MCOLN2 in Adriamycin chemoresistance, two different siRNAs (siMCOLN2-1 and siMCOLN2-2) were used to knockdown MCOLN2. Depletion of MCOLN2 significantly sensitized MCF-7/ADM cells to Adriamycin, resulting in reduced cell proliferation and decreased colony formation upon exposure to 2 µM Adriamycin (Fig. 1B-C). Additionally, by leveraging the intrinsic fluorescence of Adriamycin, we assessed intracellular drug accumulation using flow cytometry. As shown in Fig. 1D, MCOLN2 knockdown markedly increased intracellular drug levels, confirming that MCOLN2 regulates drug retention. Finally, we evaluated apoptosis using Annexin V-FITC/PI double staining. As illustrated in the supplemental Fig. 1C, Adriamycin treatment induced more robust apoptosis in MCOLN2-knockdown cells compared to control cells. In contrast, overexpression of MCOLN2 in MCF-7 cells dramatically increased the Adriamycin resistance of MCF-7 cells, as shown by the increased cell proliferation (Fig. 1E) and colony formation (Fig. 1F). The effectiveness of siMCOLN2 knockdown and MCOLN2 overexpression on MCOLN2 protein levels were confirmed by immunoblots (Fig. 1B, lower panel; Fig. 1E, upper panel). Dose-dependent cell viability curves in response to different concentration of Adriamycin were constructed. MCOLN2 silencing significantly reduced the half maximal inhibitory concentration (IC_50_) value of Adriamycin treatment in MCF-7/ADM cells (Fig. 1G), whereas exogenous expression of MCOLN2 increased the IC_50_ value in MCF-7 cells (Fig. 1H). We further investigated the role of MCOLN2 using the Adriamycin-resistant triple-negative breast cancer cell line MDA-MB-231/ADM. MCOLN2 knockdown sensitized these cells to Adriamycin, significantly suppressing their proliferation upon drug treatment. (Supplemental Fig. 1D). Together, these results demonstrate that an increased expression of MCOLN2 enhances the Adriamycin resistance in breast cancer cells.

### MCOLN2 promotes breast cancer cell autophagy

**Fig. 2:**
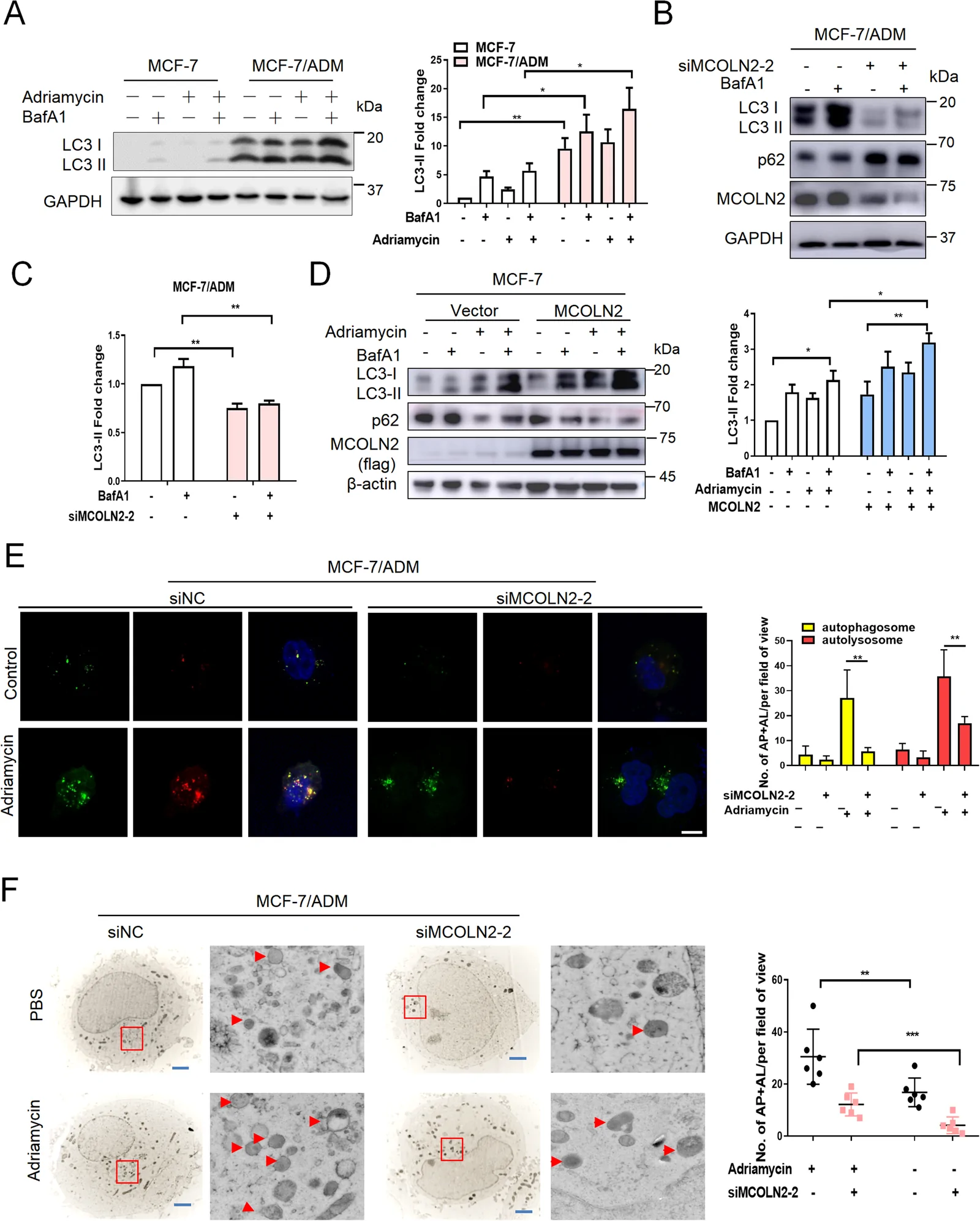
MCOLN2 promotes breast cancer cell autophagy. (**A**) MCF-7/ADM and parental MCF-7 cells were treated with or without Adriamycin (2 µM for MCF-7/ADM, 1 µM for MCF-7) for 24 h, followed by incubation with or without 100 nM BafA1 for 3 h before protein harvest. Representative immunoblot images were shown on the left and the data summary normalized to GAPDH on the right. (**B**-**C**) MCF-7/ADM cells were transfected with siMCOLN2-2 or negative control siNC. 48 h post transfection, the cells were incubated with or without 100 nM BafA1 for 3 h. Representative immunoblot images were shown in B and data summary normalized to GAPDH in C. (**D**) MCF-7 cells were transfected with MCOLN2 or empty vector plasmid. 48 h post transfection, the cells were incubated with or without 1 µM Adriamycin for 24 h, followed by incubation with or without BafA1 for 3 h before protein harvest. Representative immunoblot images were shown on the left and data summary normalized to β-actin on the right. (**E**) MCF-7/ADM cells were transfected with siMCOLN2 or siNC. The representative pictures of autophagosome (yellow dots) and autolysosomes (red dots) on the left (scale bar:10 μm) were shown and data summary on the right (*n* = 20 cells/sample examined over three independent experiments). (**F**) Transmission electron microscopy images of the MCF-7/ADM cells transfected with siMCOLN2 or siNC. The cells were treated with or without 4 µM Adriamycin for 24 h. The autophagosome and autolysosome were indicated with arrowheads on the left (scale bar:2 microns) and data summary on the right. Mean ± SD. *n* = 3 in A, C, D, E; *n* = 6 in F. *, *P* < 0.05; **, *P* < 0.01; ***, *P* < 0.001

Autophagy is an important factor prolonging the survival of cancer cells under Adriamycin chemotherapy stress [21]. We next examined whether MCOLN2 could regulate autophagy. Immunoblots were used to measure LC3-II protein accumulation level, which is a commonly used index for autophagy [22]. LC3-II level was much higher in MCF-7/ADM than in MCF-7 with or without bafilomycin A1 (BafA1) treatment (Fig. 2A), which is an inhibitor of autolysosomal fusion. Furthermore, increased LC3-II autophagic puncta was found in MCF-7/ADM cells compared with MCF-7 when these cells were treated with BafA1 or Adriamycin (Supplemental Fig. 1E-F). These results confirmed an elevated autophagy level in MCF-7/ADM than in MCF-7.

To test the effect of MCOLN2 on autophagy, we used siRNA to silence MCOLN2 and determined its effect on LC3-II protein expression. Knockdown of MCOLN2 significantly decreased LC3-II level in both MCF-7/ADM and MCF-7 cells, regardless of BafA1 treatment (Fig. 2B-C, Supplemental Fig. 2A-B). In contrast, MCOLN2 overexpression increased LC3-II levels in MCF-7 cells (Fig. 2D). Consistent with these findings, the levels of p62, a canonical autophagy receptor, were markedly elevated upon MCOLN2 knockdown and significantly reduced upon MCOLN2 overexpression (Fig. 2B and D). Because p62 bridges ubiquitinated substrates with LC3 and is degraded during autophagy, its accumulation indicates impaired autophagic degradation. Collectively, these findings demonstrate that MCOLN2 positively regulates autophagic flux. To further examine the effect of MCOLN2 on autophagic flux, we used the tandem reporter Ad-mCherry-GFP-LC3, which labeled autolysosome with red fluorescence and autophagosome with dual red and green fluorescence. As shown in Fig. 2E, knockdown MCOLN2 decreased the formation of both autophagosome (yellow in merge) and autolysosomes (red in merge) in MCF-7/ADM cells. Transmission electron microscopy (TEM) further confirmed these results. Autophagosomes, identified by their characteristic double-membrane structures enclosing undigested cytoplasmic material or intact organelles (red arrows, Fig. 2F), were reduced in MCOLN2-knockdown MCF-7/ADM cells.In addition, the RNA sequencing results of GSEA analysis showed that most of the genes that are negatively associated with autophagosome are enriched in the siMCOLN2 group (Supplemental Fig. 2C). Taken together, these results demonstrated that MCOLN2 promotes autophagy in MCF-7/ADM cells.

### MCOLN2 increases the Adriamycin resistance by promoting autophagy pathway

**Fig. 3:**
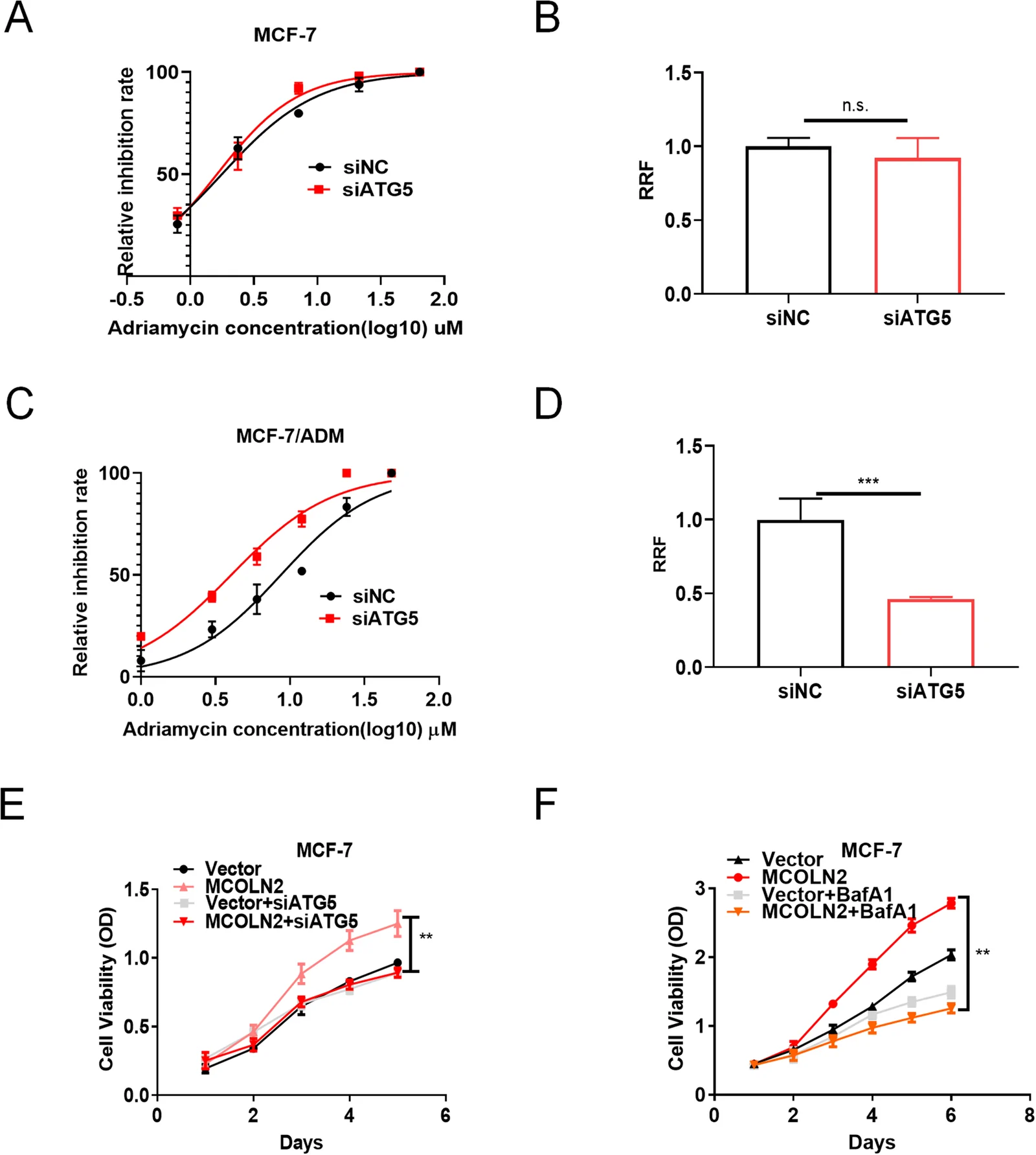
MCOLN2 increases the Adriamycin resistance by activating autophagy. (**A**-**D**) The MCF-7/ADM and the parental MCF-7 cells were transfected with siATG5. 48 h post transfection, the cells were incubated with indicated Adriamycin concentration for 48 h, followed by the CCK8 cell viability assay. B and D: averages of relative resistant factors (RRFs, the ratio of IC50 values of experimental groups relative to that of control groups) in 3–5 independent experiments. (**E**) MCF-7 cells were transfected with MCOLN2 or empty vector plasmid, and/or siATG5 and/or negative control siRNA. The cells were then incubated with 0.5 µM Adriamycin for indicated time periods. (**F**) MCF-7 cells were transfected with MCOLN2 or empty vector plasmid, followed by incubation with or without 20 nM BafA1 or PBS. The cells were then incubated with 0.5 µM Adriamycin for indicated time periods. Mean ± SD (*n* = 3–5). **, *P* < 0.01; ***, *P* < 0.001

We assessed whether autophagy is essential for Adriamycin resistance by knocking-down a critical autophagy gene ATG-5. The cells were incubated with different doses of Adriamycin for 48 h, followed cell viability assay. The results showed that knockdown of ATG-5 did not alter Adriamycin sensitivity in MCF-7 cells (Fig. 3A-B), but markedly decreased the Adriamycin resistance in MCF-7/ADM cells (Fig. 3C-D). Similarly, inhibiting autophagy with BafA1 did not alter Adriamycin sensitivity in MCF-7 cells (Supplemental Fig. 2D), but markedly decreased the Adriamycin resistance in MCF-7/ADM cells (Supplemental Fig. 2E). These data support a critical role of autophagy in enhancing Adriamycin resistance of breast cancer cells.

We further explored the relationship among MCOLN2, autophagy and Adriamycin resistance. We overexpressed MCOLN2 in MCF-7 cells, followed by incubation in 0.5 µM Adriamycin for up to 5 to 6 days. The results showed that MCOLN2 overexpression increased Adriamycin resistance compared to the control cells (Fig. 3E and F). However, MCOLN2 overexpression failed to increase the Adriamycin resistance in cells pretreated with siATG5 (Fig. 3E) or BafA1 to abolish autophagy (Fig. 3F). Together, these data support the notion that MCOLN2 acts through autophagy to enhance Adriamycin resistance.

### MCOLN2 suppresses mTOR signaling

**Fig. 4:**
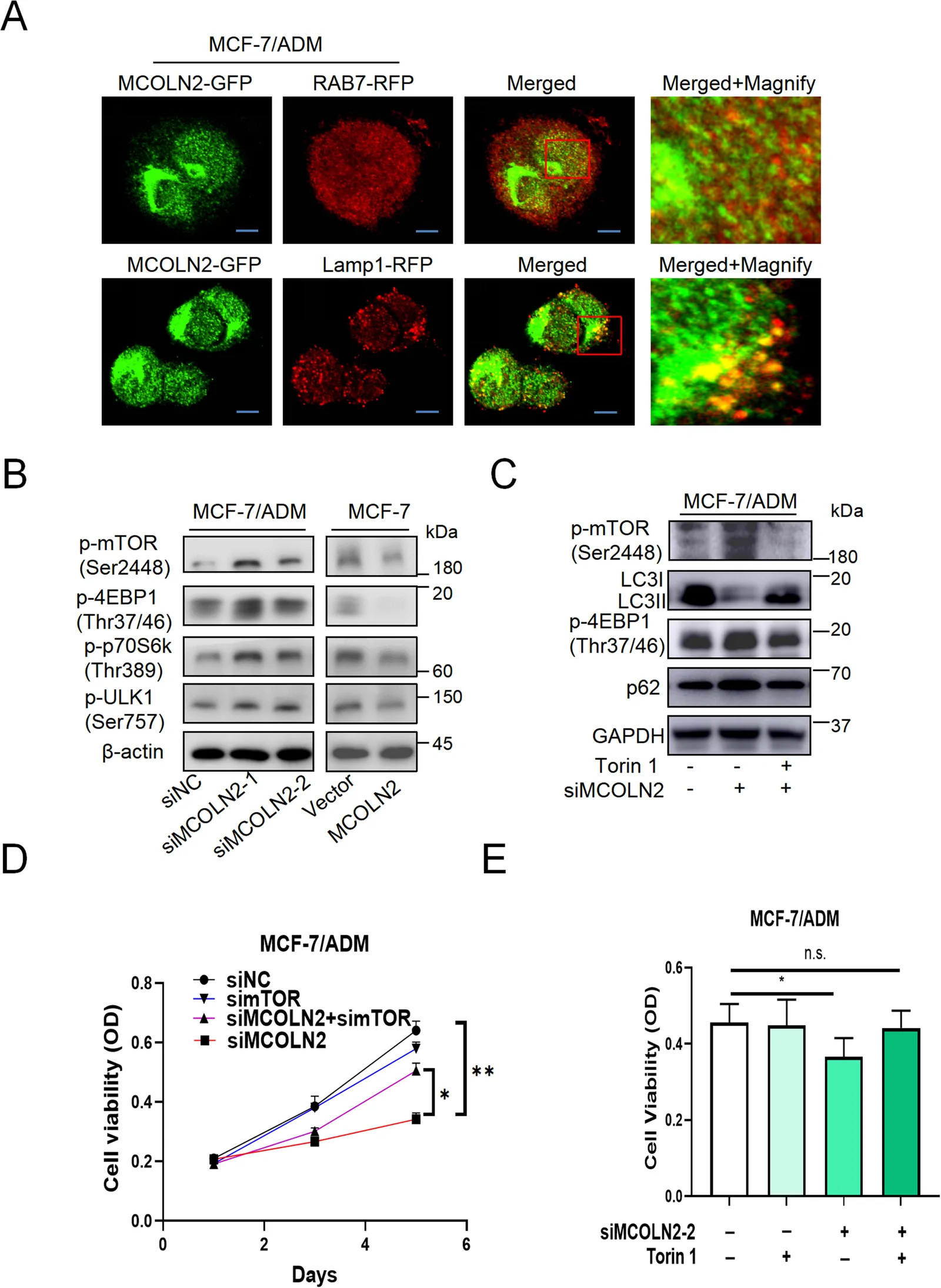
MCOLN2 suppresses mTOR signaling. (**A**) Subcellular localization of MCOLN2 in MCF-7/ADM cells. Co-localization of MCOLN2 proteins with late endosomal marker RAB7 (upper row) and lysosomal marker lamp1-RFP (bottom row). The right most panels were the magnified images of Merge. Scale bar = 10 μm. (**B**) Representative immunoblots of p-mTOR and mTORs’ substrates (p-ULK1, p-p70S6K, and p-4EBP1) in MCF-7/ADM cells transfected with siMCOLN2 or siNC, and MCF-7 cells transfected with MCOLN2 or empty vector plasmid. (**C**) The expression levels of p-mTOR (Ser2448), LC3I/II, p-4EBP1 (Thr37/46), and p62 were detected in MCF-7/ADM cells treated with Torin1 (±) and siMCOLN2 (±). GAPDH was used as an internal control. (**D**) MCF-7/ADM cells were co-transfected with siMCOLN2 and/or si-mTOR, then incubated with Adriamycin for indicated time periods. The relative cell viability was monitored by CCK8 assays. (**E**) The MCF-7/ADM cells transfected with siMCOLN2 or siNC were pretreated with 10 µM Torin1 or DMSO for 1 h. The cells were then treated with 4 µM Adriamycin for 48 h, followed by the CCK8 cell viability assay. Mean ± SD (*n* = 3). n.s, not significant; *, *P* < 0.05; **, *P* < 0.01; ***, *P* < 0.001

It’s well known that mTOR signaling pathway negatively regulates autophagy, and that the mTOR complex are located and activated on the lysosome [23]. Because MCOLN2 is an endolysosomal Ca^2+^ channel [10], we explored the possible role of MCOLN2 in regulating mTOR. The endolysosomal localization of MCOLN2 in MCF-7/ADM cells was validated by its colocalization with late endosomal marker RAB7 and lysosomal marker Lamp1 (Fig. 4A). To test whether MCOLN2 could regulate the mTOR signaling pathway, we knockdown MCOLN2 by two siRNAs (siMCOLN2-1 and siMCOLN2-2). Both siMCOLN2 increased the phosphorylation levels of mTOR (Ser2448) and its substrates (p70S6k-Thr389, ULK1-Ser757, and 4EBP1-Thr37/46) in MCF-7/ADM cells (Fig. 4B). In contrast, overexpression of MCOLN2 significantly reduced phosphorylation levels of mTOR (Ser2448) and its substrates (p70S6k-Thr389, ULK1-Ser757, and 4EBP1-Thr37/46) in its parental MCF-7 cells (Fig. 4B). To confirm that MCOLN2 regulates autophagy specifically via the mTOR pathway, we pharmacologically inhibited mTOR using Torin1 in MCOLN2-knockdown cells. As shown in Fig. 4C, MCOLN2 knockdown significantly decreased LC3-II levels and increased p62 accumulation, concomitant with elevated phosphorylation levels of mTOR and its substrates 4EBP1(Thr37/46), indicating autophagy suppression. Notably, treatment of these knockdown cells with Torin1 effectively rescued LC3-II expression and restored p62 degradation. These results demonstrate that mTOR acts as the critical mediator linking MCOLN2 to autophagy activation. Similarly, MCOLN2 knockdown in MDA-MB-231/ADM cells also increased p62 levels and mTOR (Ser2448) phosphorylation, while reducing LC3-II accumulation (Supplemental Fig. 3A). These data further confirm that MCOLN2 suppresses mTOR activity in breast cancer cells. We next explored whether the regulation of mTOR by MCOLN2 can affect the Adriamycin resistance. As expected, knockdown of MCOLN2 by siMCOLN2 reduced the Adriamycin resistance. However, in the cells where mTOR was either knocked down by si-mTOR or inhibited by Torin1, siMCOLN2 failed to reduce the Adriamycin chemoresistance (Fig. 4D-E, supplemental Fig. 3B). As controls, we verified that si-mTOR could indeed effectively knockdown the mTOR protein expression (Supplemental Fig. 3C). Taken together, these data strongly support the notion that MCOLN2 acts through mTOR to enhance Adriamycin resistance.

### MCOLN2 promotes mTOR degradation

**Fig. 5:**
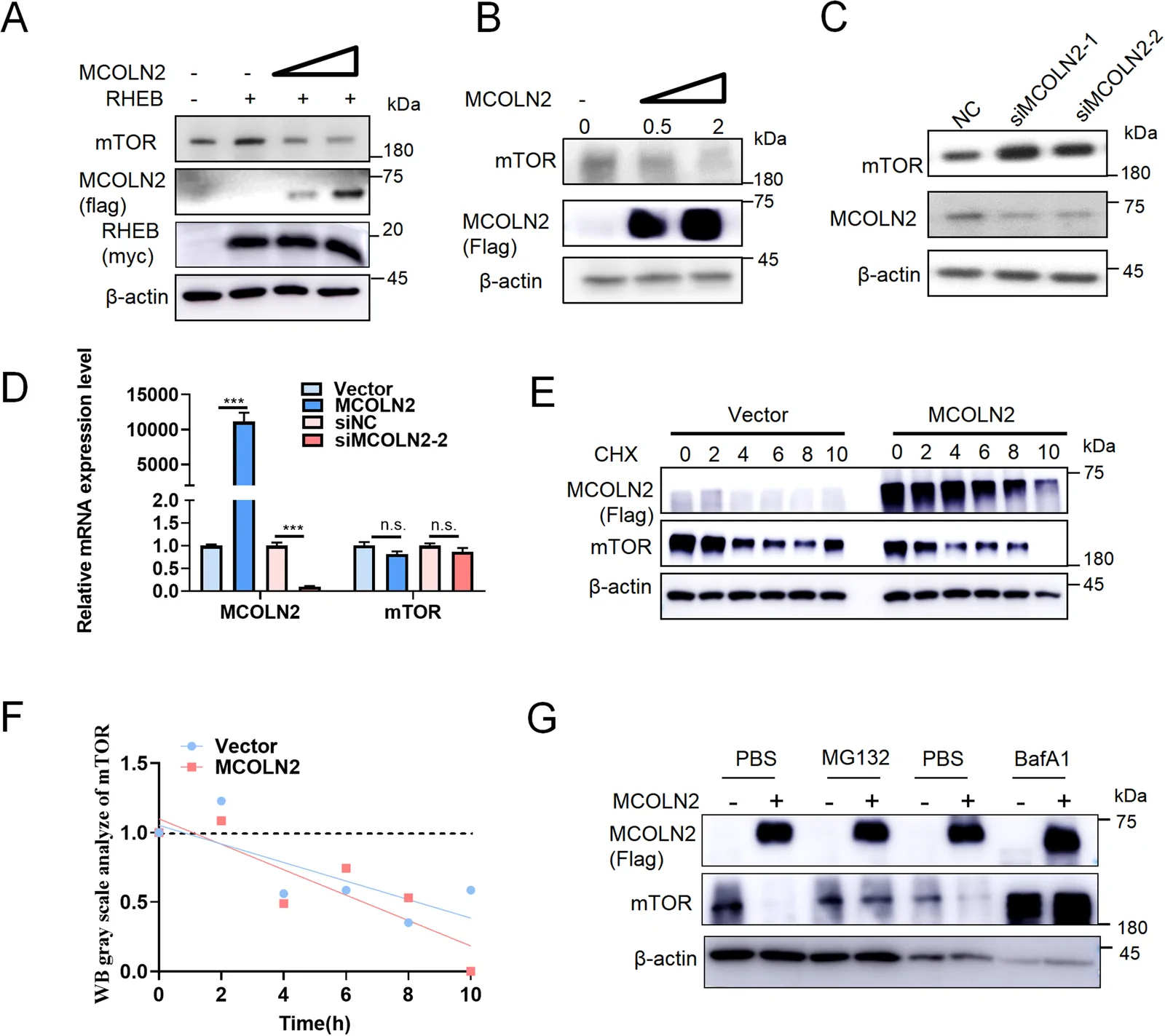
MCOLN2 promotes mTOR degradation. (**A**-**B**) Dose-dependent overexpression of flag-MCOLN2 plasmid in suppressing mTOR protein levels in myc-RHEB-expressing (**A**) or control MCF-7 cells (**B**). (**C**) Knockdown of MCOLN2 by two different siMCOLN2 increases the mTOR protein level in MCF-7/ADM cells. (**D**) Quantitative RT-PCR analysis of mTOR mRNA levels in MCF-7/ADM cells transfected with siNC or siMCOLN2 siRNA, and in MCF-7 cells transfected with flag-MCOLN2 or empty vector plasmid. (**E**-**F**) Cycloheximide chase assay comparing mTOR protein degradation between the HEK293T cells transfected with MCOLN2 or empty vector plasmid. Shown were representative immunoblots (**E**) and quantification (**F**). (**G**) MG132 or BafA1 treatment for 6 h each inhibited the MCOLN2 overexpression-induced mTOR protein degradation in MCF-7 cells. *n* = 3; Mean ± SD. n.s, not significant; ***, *P* < 0.001

To further determine whether MCOLN2-mediated autophagy is mTOR-dependent, we overexpressed RHEB-myc to activate mTOR in MCF-7 cells [24]. As a result, compared to the empty vector controls, RHEB partially restore mTOR activity, as shown by the increased phosphorylation of ULK1 and p70S6k (Supplemental Fig. 3D). Furthermore, we found that overexpression of MCOLN2 dose-dependently reduced the mTOR protein level in these cells (Fig. 5A). To verify this, we also overexpressed MCOLN2 in MCF-7 cells without RHEB-myc. Similarly, overexpression of MCOLN2 dose-dependently reduced the mTOR protein level in MCF-7 cells (Fig. 5B), whereas knockdown of MCOLN2 by siMCOLN2 increased the mTOR protein level in MCF-7/ADM cells (Fig. 5C). Intriguingly, quantitative RT-PCR analysis showed that neither MCOLN2 overexpression nor MCOLN2 knockdown had any effect on mTOR mRNA levels (Fig. 5D). Therefore, we speculate that MCOLN2 may regulate mTOR at the level of protein synthesis or degradation rather than at mRNA level.

Next, protein turnover assays were used to determine the mTOR degradation/stability in the presence of cycloheximide, which abolishes the protein synthesis. The results showed that MCOLN2 overexpression accelerated time-dependent mTOR protein degradation (Fig. 5E and F). To further determine whether MCOLN2-mediated mTOR degradation was through lysosome or ubiquitin-proteasome system, we utilized a lysosome inhibitor BafA1 and a proteasome inhibitor MG132. The results demonstrated that MCOLN2 mediated mTOR degradation is mostly mediated via lysosomal degradation pathway and partly via proteasome degradation pathway (Fig. 5G). Furthermore, in the presence of BafA1, the MCOLN2 overexpression failed to induce mTOR protein degradation (Fig. 5G), suggesting a critical role of lysosomal degradation in MCOLN2-mediated mTOR degradation.

### MCOLN2 knockdown reverses the Adriamycin resistance in vivo

**Fig. 6:**
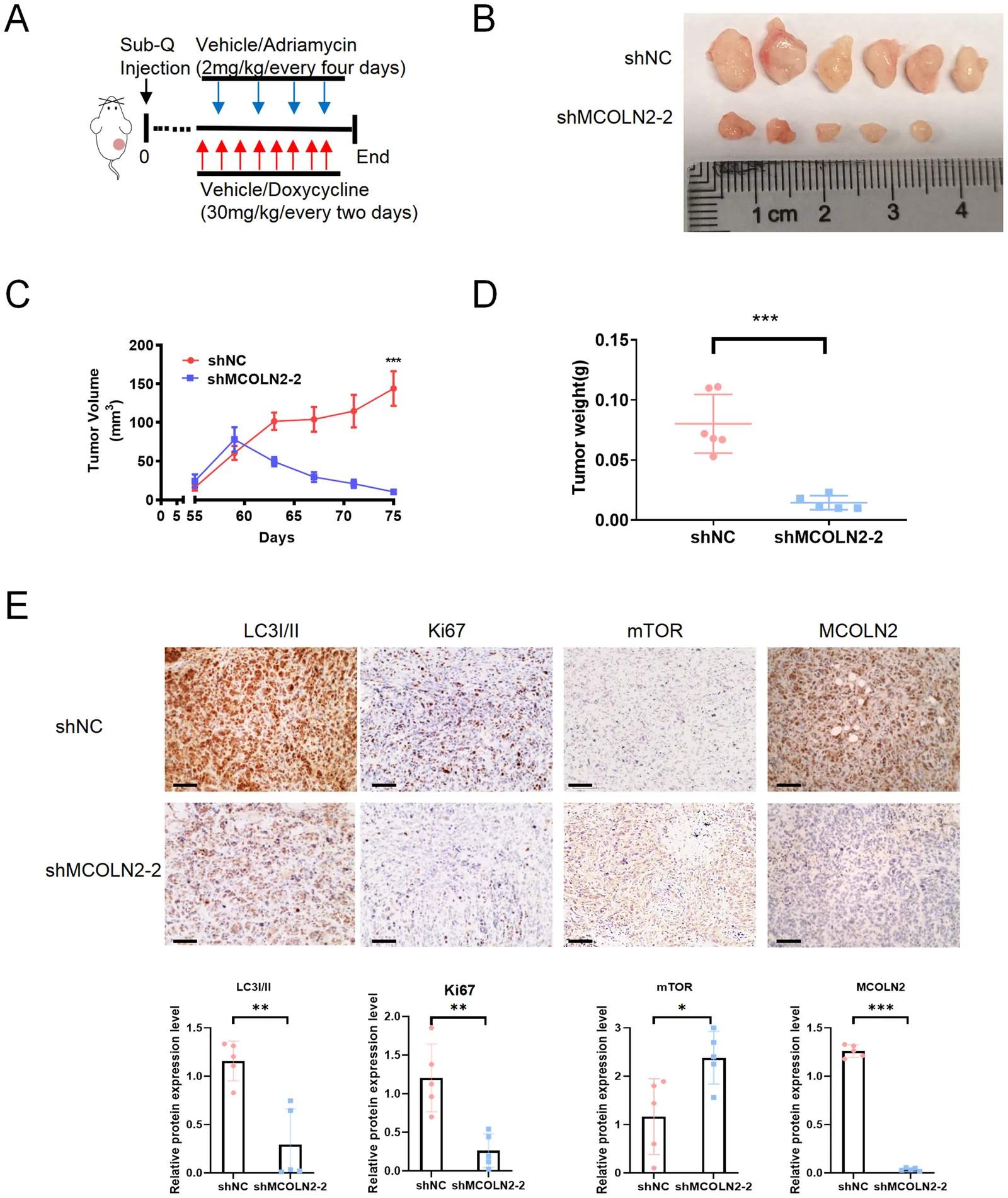
MCOLN2 knockdown reverses the Adriamycin resistance in vivo. (**A**) Schematic drawing of doxycycline and Adriamycin administration. (**B**) The effect Dox-inducible MCOLN2 knockdown by shMCOLN2-2 on the growth of tumor xenografts under Adriamycin in NCG mice. Shown were the images of xenograft tumors harvested at the end of the experiment. (**C**) Growth curves of tumor xenografts. (**D**) The weights of tumors at end point are presented as a dot plot. (**E**) Representative IHC images assessing LC3-I/II, Ki67, mTOR and MCOLN2. Mean ± SD, Scale bar = 100 μm (*n* = 5/group in **E**); *, *P* < 0.05; **, *P* < 0.01; ***, *P* < 0.001

To investigate the role of MCOLN2 in regulating Adriamycin resistance of breast cancer in vivo, doxycycline (Dox)-inducible MCOLN2 knockdown cells (shMCOLN2-1 and shMCOLN2-2) were inoculated into the lower back of immunodeficient NCG mice. The mice were treated with Adriamycin when tumors became palpable. Consistent with the results in vitro, Adriamycin treatment shrunk the tumors much more in MCOLN2 knockdown group than in control group, suggesting that knockdown of MCOLN2 by shMCOLN2-1 (Supplemental Fig. 4A-C) and shMCOLN2-2 (Fig. 6A-D) restored the Adriamycin sensitivity in Adriamycin-resistant tumors. Immunohistochemical staining (IHC) showed that the expression levels of Ki67 and LC3-I/II were markedly lower while mTOR expression level was higher in the MCOLN2 knockdown group than in control group (Fig. 6E). Taken together, these results support the notion that MCOLN2 knockdown increases the mTOR protein level, inhibits autophagy, and reverses the Adriamycin resistance in vivo.

### MCOLN2 expression is elevated in chemoresistant breast cancer patients and associated with poor prognosis

**Fig. 7:**
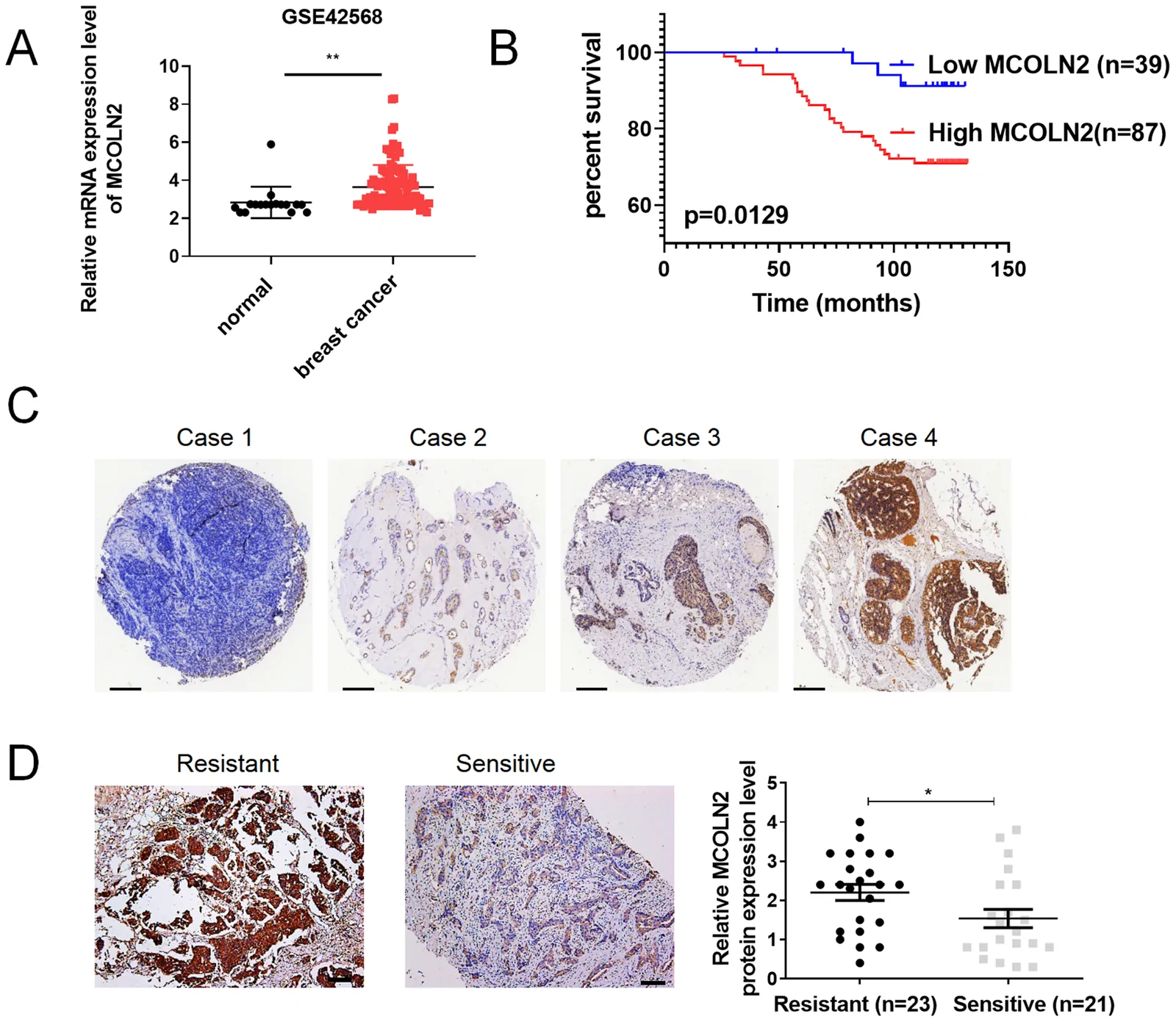
MCOLN2 expression is elevated in chemoresistant breast cancer patients and associated with poor prognosis. (**A**) Comparison of MCOLN2 expression between breast tumor samples and normal breast tissues using GSE42568 cohort from Gene Expression Omnibus datasets. (**B**) Kaplan-Meier plot of overall survival time and log-rank test based on MCOLN2 expression of breast cancer tissue microarray (TMA). (**C**) Representative MCOLN2 staining images of breast cancer patients’ samples at different expression level. The staining intensity and percentage were analyzed by Halo pathology software. Scale bar = 250 μm. (**D**) Representative IHC images of MCOLN2 expression in breast cancer tissue with poor chemotherapy response (resistant) or good chemotherapy response (sensitive). The staining intensity and percentage were analyzed by Halo pathology software. Unpaired student’s t test was performed. Scale bar = 100 μm *, *P* < 0.05; **, *P* < 0.01

Bioinformatical studies were performed to evaluate clinically relevance of our findings. GEO database analysis showed that MCOLN2 was significantly upregulated in breast cancer tissues compared to normal tissues (Fig. 7A). To determine the prognostic value of MCOLN2, the data from a large-scale human breast cancer tissue microarray (TMA) were used for Kaplan–Meier analysis. Of note, the breast cancer patients with higher MCOLN2 expression have significant poor prognosis compared to those with lower MCOLN2 expression (Fig. 7B-C). We further analyzed the expression of MCOLN2 proteins in tumor sections of 44 breast cancer patients after chemotherapy. Treatment response was assessed by the response evaluation criteria in solid tumors according to Miller-Payne grading system [18]. Among 44 patients, 21 patients responded to chemotherapy whose Miller-Payne grading were 4 or 5 (good outcome or chemotherapy-sensitive), whereas 23 patients were resistant to the chemotherapy whose grade were 1 or 2 (poor outcome or chemoresistant). The IHC results showed that MCOLN2 expression level was much higher in chemoresistant tissues than in chemosensitive tissues (Fig. 7D).

## Discussion

**Fig. 8:**
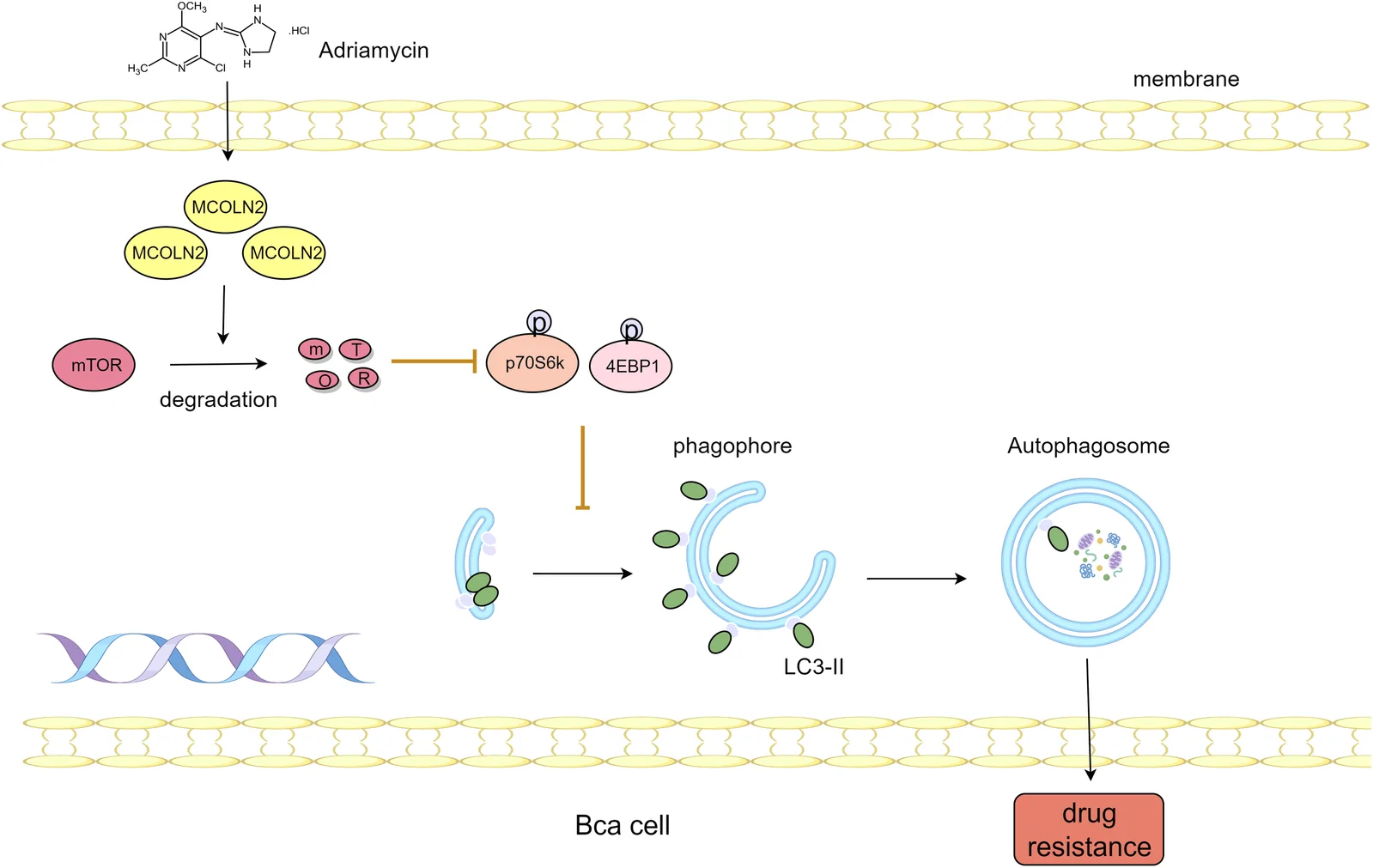
Schematic illustration of the mechanism underlying MCOLN2-mediated Adriamycin resistance. In the context of Adriamycin resistance, breast cancer cells exhibit upregulated MCOLN2 expression. This upregulation promotes the lysosomal degradation of the mTOR protein. Consequently, the phosphorylation levels of downstream mTOR targets are decreased, which in turn promotes autophagosome formation and enhances cancer cell survival under Adriamycin treatment. In this schematic, yellow bars indicate inhibitory events, while arrows represent activation or promotion. (Created with FigDraw)

Systematic and neoadjuvant Adriamycin-based chemotherapy is a common treatment strategy for advanced breast cancer according to NCCN guidelines [25]. However, patients who do not respond to Adriamycin treatment suffer, rather than benefit from chemotherapy. Thus, identifying new mechanisms for Adriamycin resistance is critical for developing personalized and precise treatments for breast cancer patients. Ca^2+^ is an important second messenger that participates in the regulation of cancer cell chemoresistance [8, 9]. Most of previous chemoresistance-related studies have been focused on the Ca^2+^-permeable channels localized on the plasma membrane [8, 9]. For example, we previously reported an important role of TRPC5 in Adriamycin resistance to breast cancer cells [8]. Only very recently, researchers began to explore the possible role of endolysosomal MCOLN/TRPML channels in cancer chemoresistance. In this regard, MCOLN1 is found to increase chemoresistance in leukemia and ovarian cancer [26, 27]; MCOLN2 enhances chemoresistance in glioma [15]; MCOLN3 contributes to chemoresistance in non-small cell lung cancer [28]. However, none of these previous studies explored the role of MCOLN/TRPML in the chemoresistance of breast cancer. In the present study, we found that MCOLN2 is upregulated in chemoresistant breast cancer samples, which correlates with poor prognosis in breast cancer patients. More importantly, knockdown of MCOLN2 reduced the Adriamycin chemoresistance of MCF-7/ADM cells in vitro and in vivo, while overexpression of MCOLN2 promoted the Adriamycin chemoresistance of MCF-7 cells in vitro. These results, for the first time, demonstrated a critical role of MCOLN2 in enhancing chemoresistance of breast cancer, highlighting an exciting possibility of targeting MCOLN2 in overcoming chemoresistance in breast cancer (Fig. 8).

Autophagy is a tightly regulated catabolic process that facilitates nutrient recycling from damaged cellular components/organelles through lysosomal degradation [3]. Substantial evidence has indicated that autophagy plays a key regulatory role in the regulation of chemoresistance in breast cancer [3]. MCOLN/TRPML channels mediate endolysosomal Ca^2+^ release and participate in the control of lysosomal degradation [29]. Therefore, it is conceivable that MCOLN/TRPML channels may regulate autophagy. Indeed, MCOLN1 and MCOLN3 are reported to modulate autophagic progress. One study shows that MCOLN1 exhibits high expression in triple-negative breast cancer (TNBC), where it stimulates the growth of TNBC via promoting mTORC1 activity and facilitating TNBC cell invasion through increasing lysosomal ATP release [30]. Another study showed that MCOLN1 promotes the proliferation, survival and tumor growth of human melanoma, but here MCOLN1 negatively regulates mTORC1 and MAPK pathway [31]. These data suggest that MCOLN1 may have a bidirectional effect on regulating mTOR pathway dependent upon cancer cell types and/or the stress that the cells are facing. As for MCOLN3, studies found that MCOLN3 regulates intracellular membrane trafficking to promote autophagy [32, 33]. MCOLN3 could also regulate autophagosome biogenesis through the interaction with the ATG8 homologue GATE16 [34]. To our knowledge, there is still no report exploring the role of MCOLN2 in autophagy. In the present study, several lines of evidence strongly support the notion that MCOLN2 promotes autophagy in estrogen receptor-positive breast cancer cells: (1) Immunoblots showed that knockdown of MCOLN2 decreased the LC3-II level, while overexpression of MCOLN2 had opposite effect; (2) Tandem reporter Ad-mCherry-GFP-LC3 analysis showed that knockdown of MCOLN2 decreased the formation of both autophagosome and autolysosomes in MCF-7/ADM cells; (3) Transmission electron microscopy data confirmed that MCOLN2 knockdown decreased the number of autophagosomes in MCF-7/ADM cells.

We further explored the mechanism of how MCOLN2 promotes autophagy. Knockdown of MCOLN2 was found to increase mTOR activity, while overexpression of MCOLN2 reduced the mTOR activity, as indicated by phosphorylation levels of mTOR and its downstream targets. Intriguingly, cycloheximide chase assay demonstrated that overexpression of MCOLN2 can promote the degradation of mTOR proteins. Together, these data for the first time demonstrated that endolysosomal MCOLN/TRPML channels can decrease mTOR stability to promote autophagy flux.

In summary, MCOLN2 is up-regulated in Adriamycin-resistant cells and chemoresistant breast cancer tissues. MCOLN2 up-regulation is associated with poor prognosis in breast cancer patients and outcome of chemo-radiotherapy. Mechanistically, MCOLN2 decreases mTOR stability to promote autophagy, subsequently increasing the Adriamycin resistance in breast cancer cells. These findings indicate that MCOLN2 may serve as a predictive biomarker in the development of personalized and precise treatment strategies and that MCOLN2 could be a treatment target to reverse chemotherapy resistance.

## Supplementary Information

Below is the link to the electronic supplementary material.

Supplementary Material 1

Supplementary Material 2

Supplementary Material 3

## Data Availability

All data supporting the findings of this study are included in the article and its supplementary materials. Additional raw data, including uncropped Western blots, qPCR Ct values, and original microscopy images, are available from the corresponding author upon reasonable request.
