# Supplementary material for: MCOLN2 (Mucolipin-2) promotes autophagy to confer Adriamycin resistance in breast cancer by regulating mTOR degradation
Source: Cell Oncol (Dordr). 2026 Aug 3;49(6):141. doi: 10.1007/s13402-026-01270-x (PMC13719976; doi:10.1007/s13402-026-01270-x)

figure 1

A

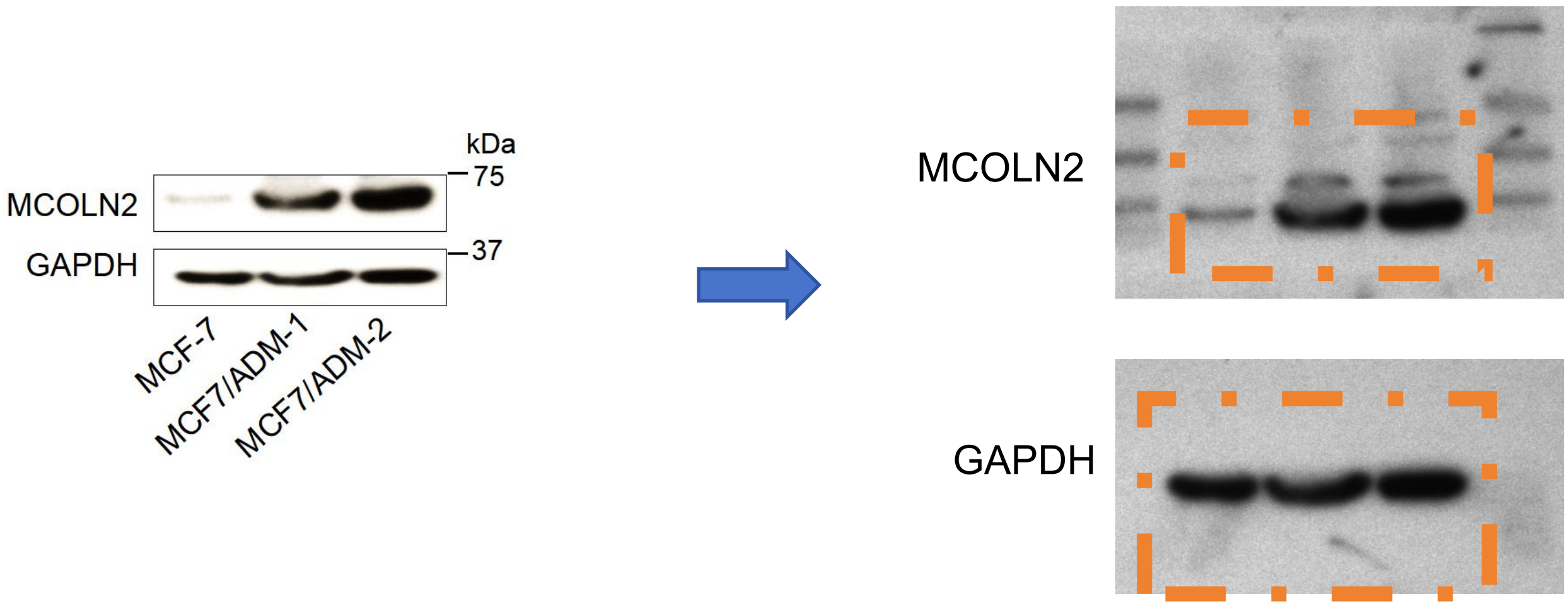
kDa
75
MCOLN2
GAPDH
37
MCF-7
MCF7/ADM-1
MCF7/ADM-2
MCOLN2
GAPDH

B

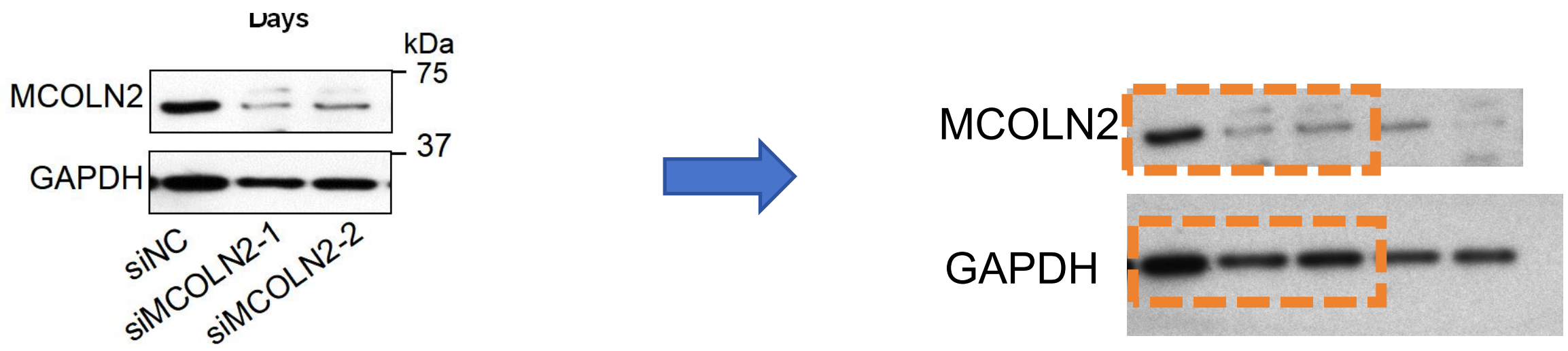
Days
kDa
75
MCOLN2
GAPDH
37
siNC
siMCOLN2-1
siMCOLN2-2
MCOLN2
GAPDH

E

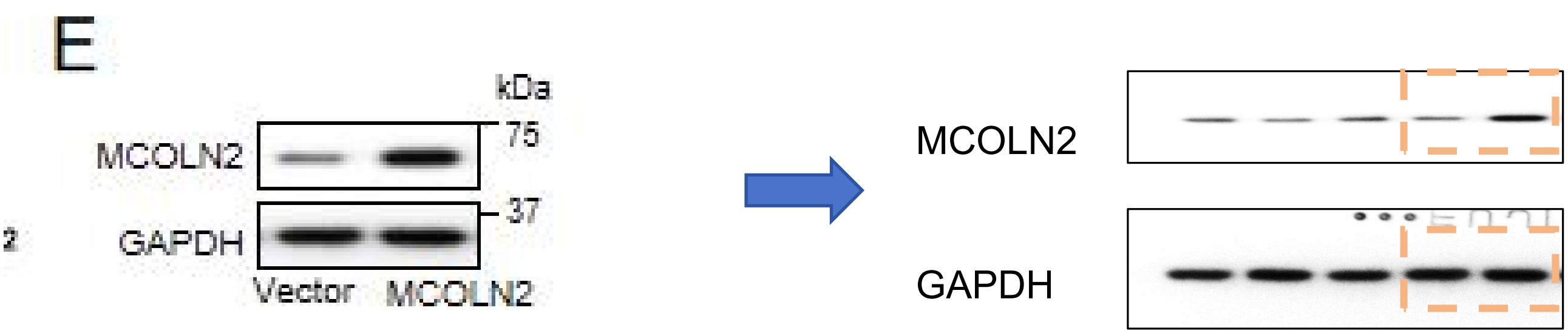
E
kDa
MCOLN2
75
GAPDH
37
Vector
MCOLN2
MCOLN2
GAPDH

figure 2

A

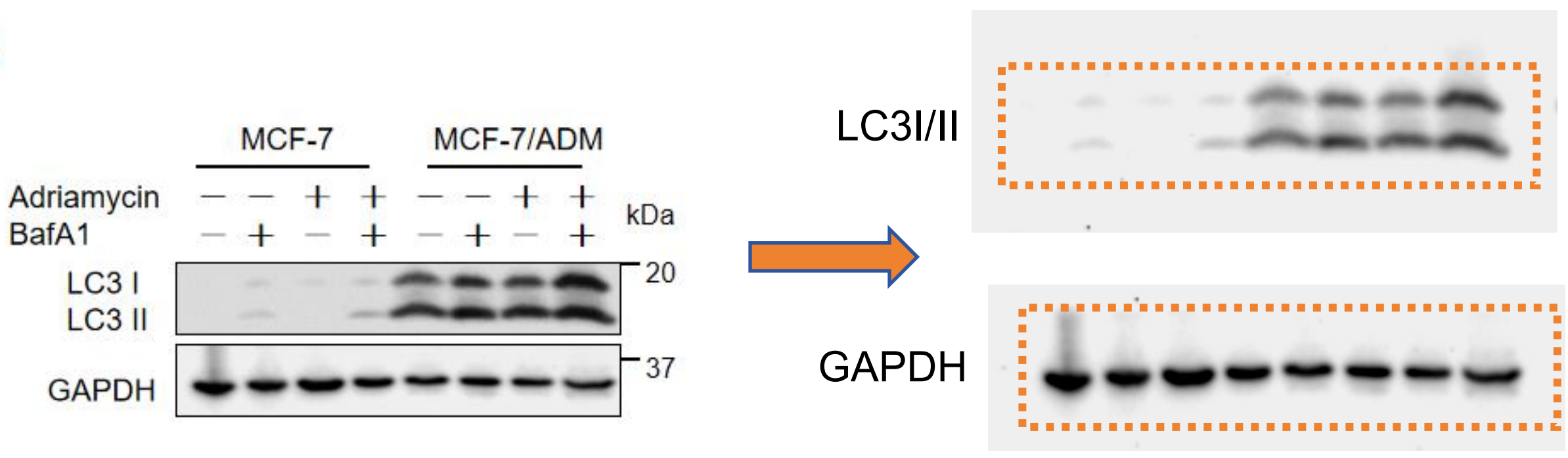
MCF-7
MCF-7/ADM
Adriamycin
BafA1
LC3 I
LC3 II
GAPDH
kDa
20
37
LC3I/II
GAPDH

B

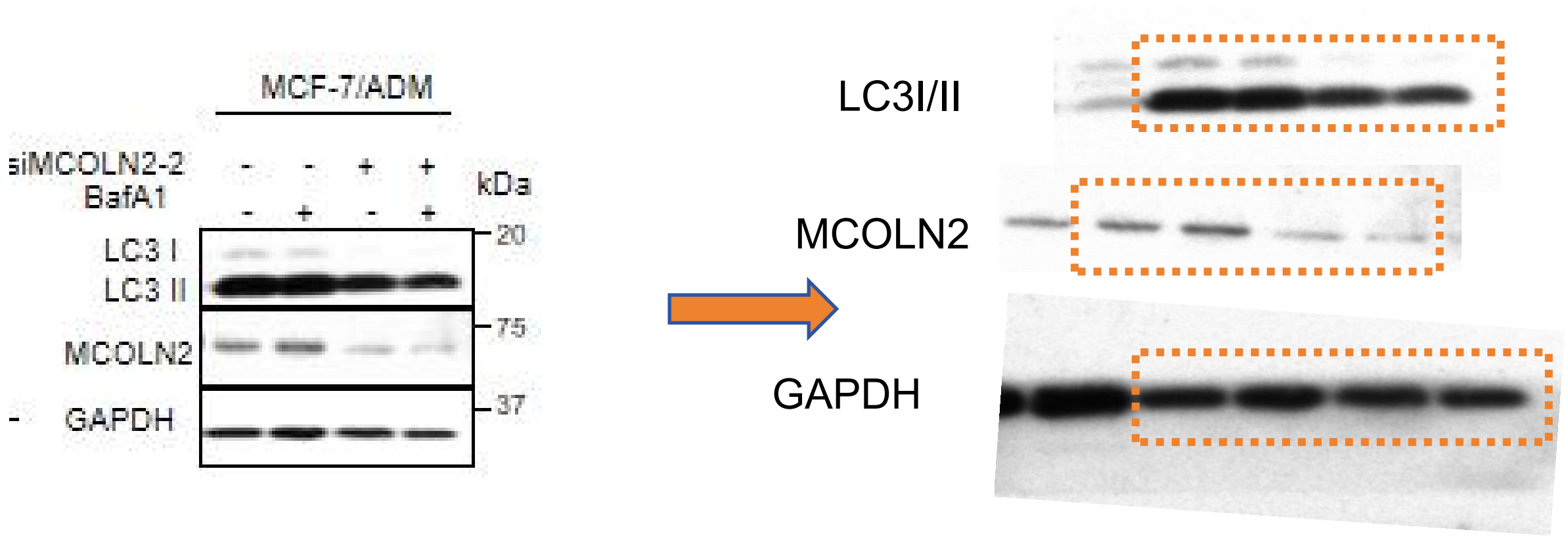
MCF-7/ADM
siMCOLN2-2
BafA1
LC3 I
LC3 II
MCOLN2
GAPDH
kDa
20
75
37
LC3I/II
MCOLN2
GAPDH

D

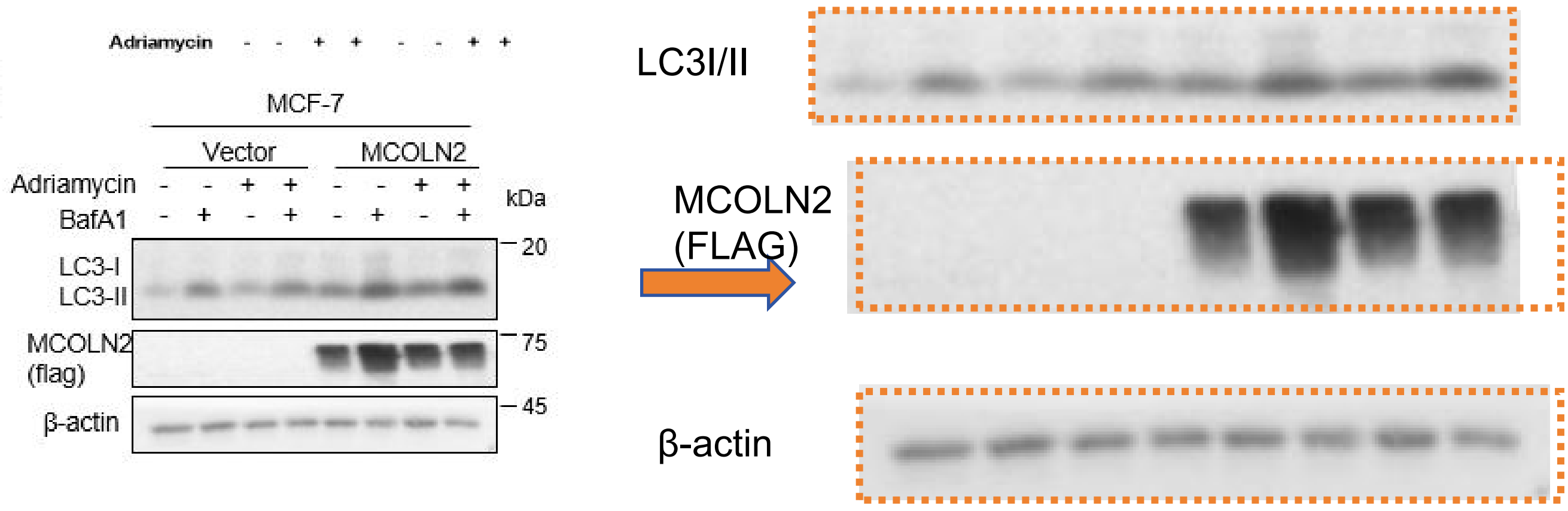
Adriamycin
MCF-7
Vector
MCOLN2
Adriamycin
BafA1
LC3-I
LC3-II
MCOLN2
(flag)
β-actin
kDa
20
75
45
LC3I/II
MCOLN2
(FLAG)
β-actin

figure 4

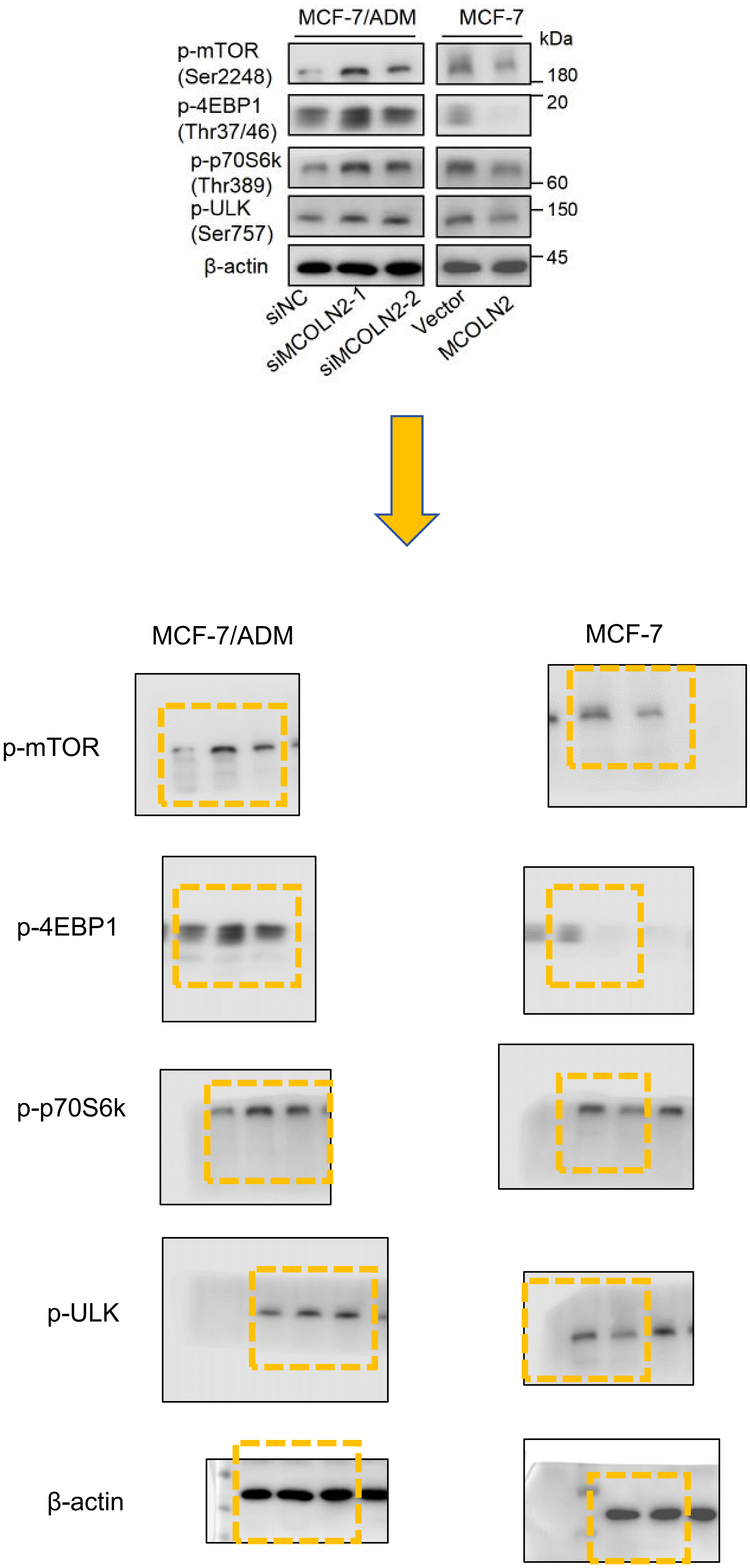

MCF-7/ADM
MCF-7
kDa
p-mTOR (Ser2248)
180
p-4EBP1 (Thr37/46)
20
p-p70S6k (Thr389)
60
p-ULK (Ser757)
150
β-actin
45
siNC
siMCOLN2-1
siMCOLN2-2
Vector
MCOLN2
MCF-7/ADM
MCF-7
p-mTOR
p-4EBP1
p-p70S6k
p-ULK
β-actin

figure 5

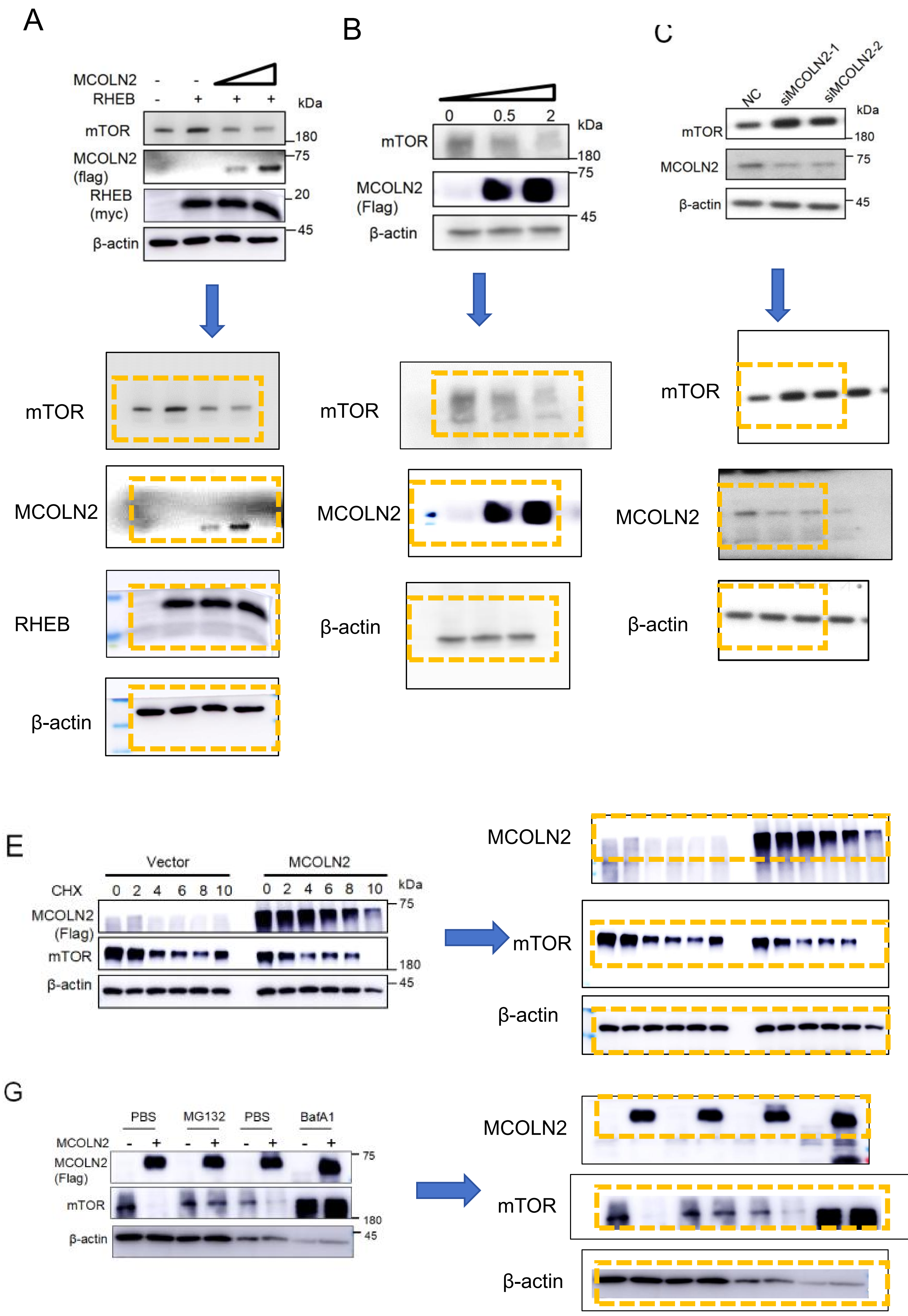

A
MCOLN2
RHEB
kDa
mTOR
180
MCOLN2 (flag)
75
RHEB (myc)
20
β-actin
45
B
0
0.5
2
kDa
mTOR
180
75
MCOLN2 (Flag)
45
β-actin
C
NC
siMCOLN2-1
siMCOLN2-2
kDa
mTOR
180
MCOLN2
75
β-actin
45
mTOR
MCOLN2
RHEB
β-actin
E
Vector
MCOLN2
CHX
0 2 4 6 8 10
0 2 4 6 8 10
kDa
MCOLN2 (Flag)
75
mTOR
180
β-actin
45
G
PBS
MG132
PBS
BafA1
MCOLN2
MCOLN2 (Flag)
75
mTOR
180
β-actin
45

Supplemental figure 1

D

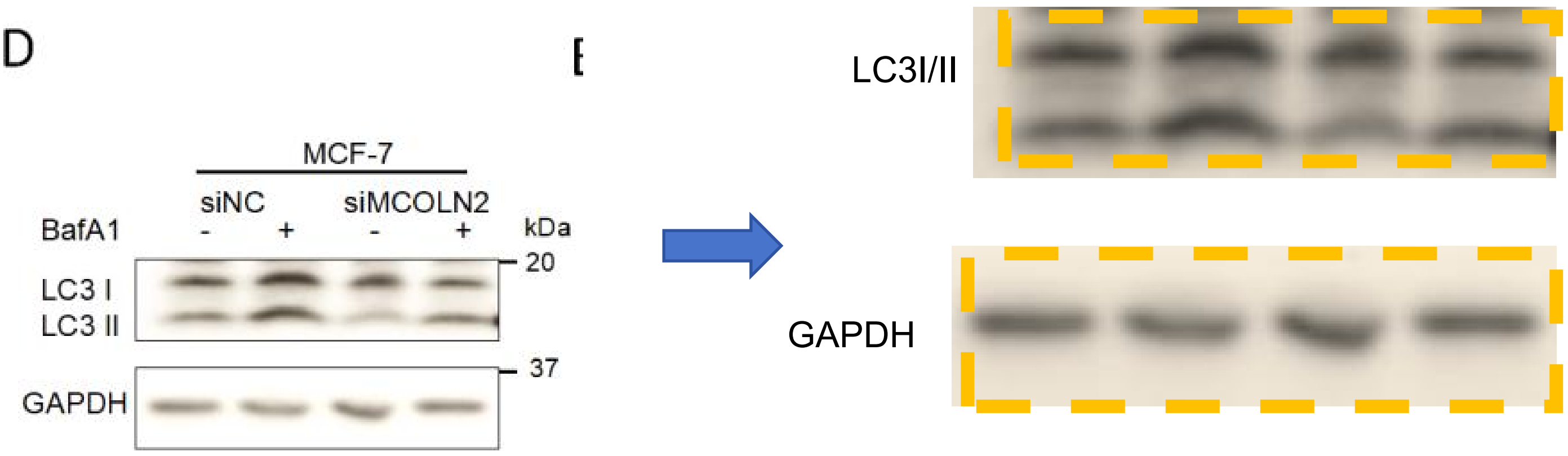

G

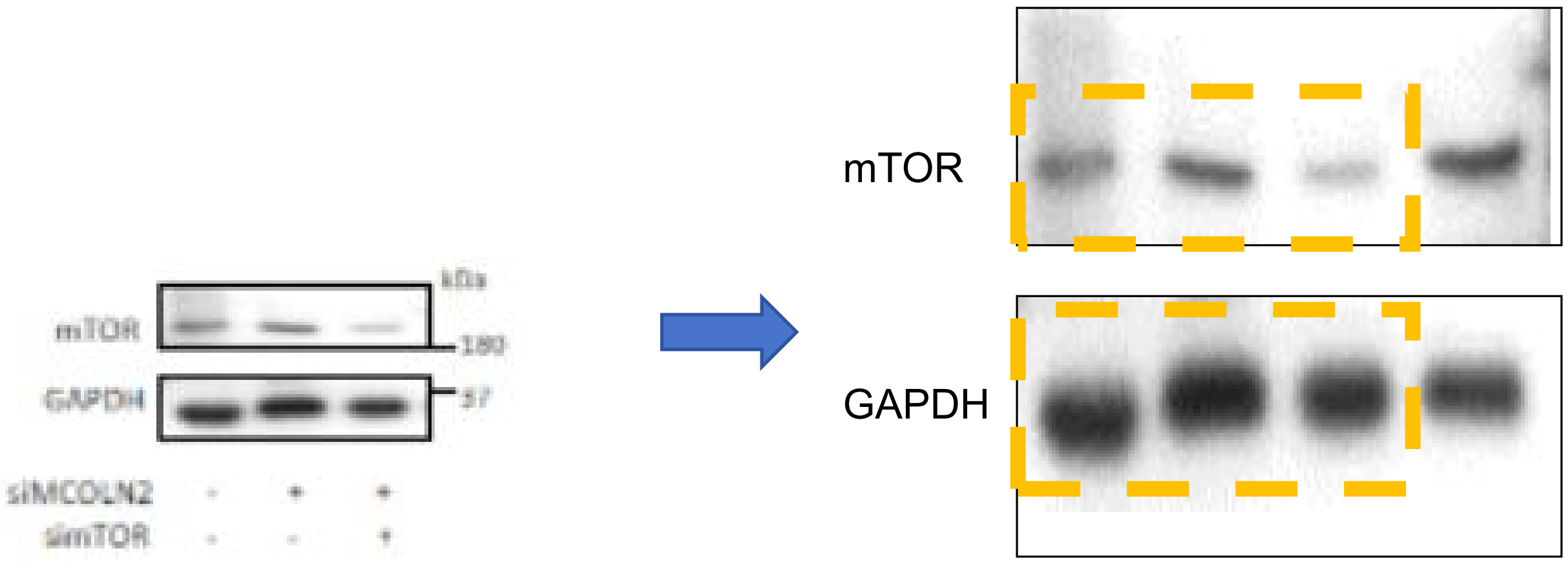

Supplemental figure 2

C

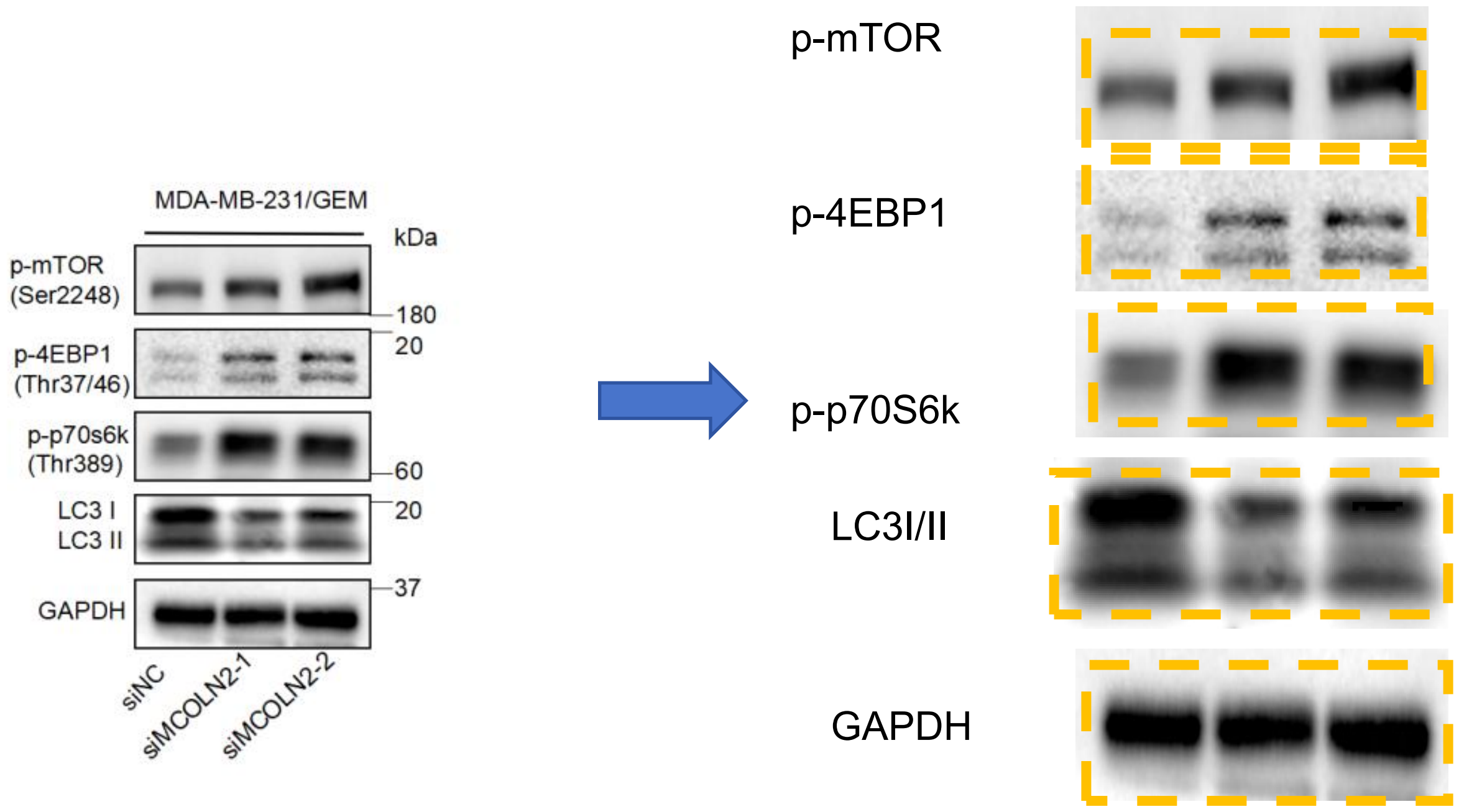

D

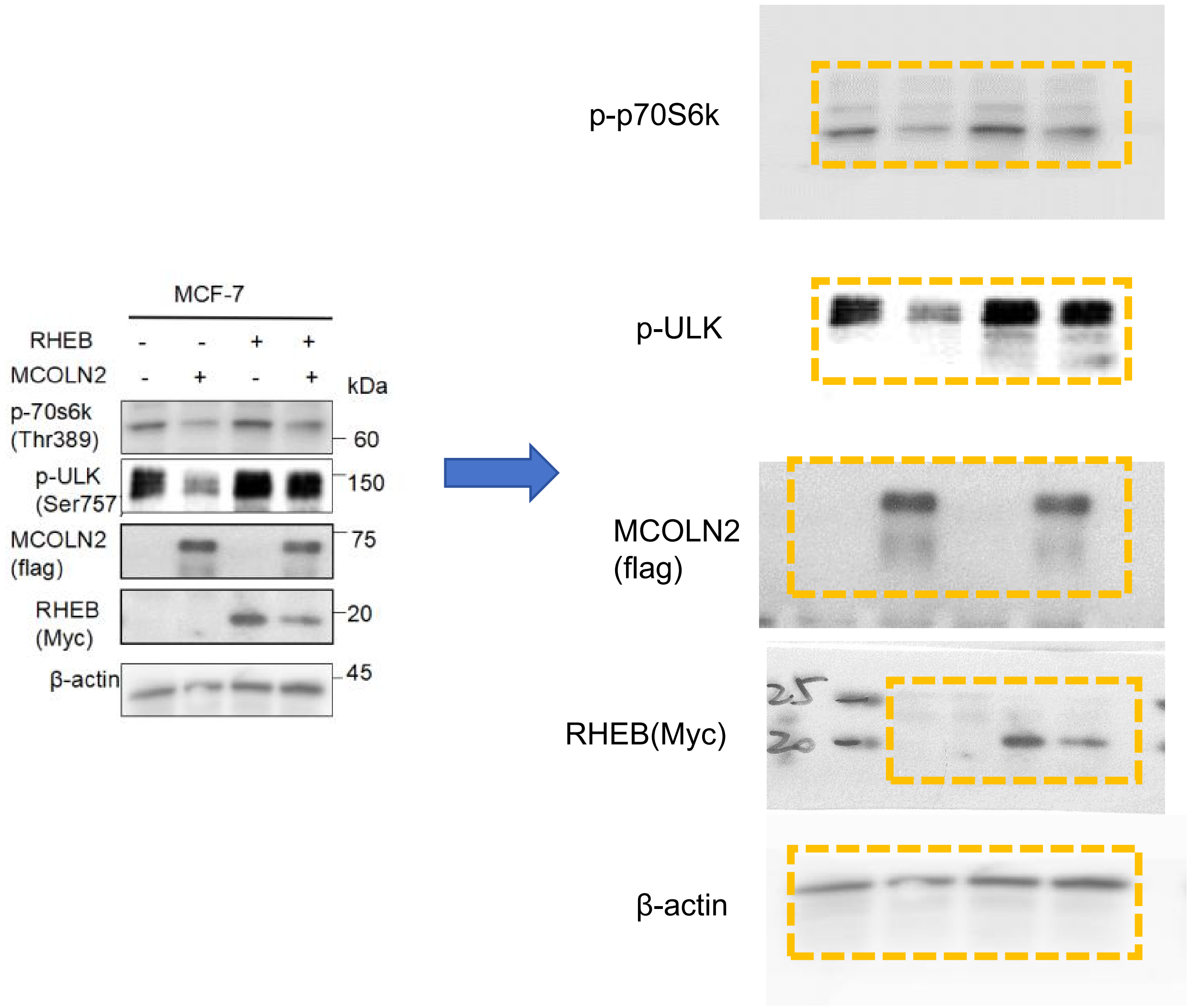

Supplement: Supplementary file 2 [file 13402_2026_1270_MOESM2_ESM.pdf]
