# Supplementary material for: MCOLN2 (Mucolipin-2) promotes autophagy to confer Adriamycin resistance in breast cancer by regulating mTOR degradation
Source: Cell Oncol (Dordr). 2026 Aug 3;49(6):141. doi: 10.1007/s13402-026-01270-x (PMC13719976; doi:10.1007/s13402-026-01270-x)
Supplement: Supplementary file 3 [file 13402_2026_1270_MOESM3_ESM.pdf]

Table S1. Primer sequence of RT-PCR

Genes	Sense primer	Antisense primer
MCOLN2	CGGCAGCCTTATCGTTTTCC	CCATTGCGTTTCTGACGGTTA
MCOLN1	GAGTCCCTGCGACAAGTTTC	TTCTCTTCCCGGAATGTC
MCOLN3	CTGCTGTGCAGCTATGATTTAC	GGAAGCCATCTTGTTGGTATTG
mTOR	GACCTTCTGCCTTCACAGATAC	TTGCCTTCTGCCTCTTATGG
GAPDH	AAGGTGAAGGTCGCACTCAA	GGGGTCATTGATGGCAACAATA

Table s2 The oligonucleotides sequence for Dox inducible shRNAs against human MCOLN2

MCOLN2 shRNA #1	CCGGGCTCGATTGTGTTCAATTACTCGAGTAATTGAACACAATCGAGCTTTTTG(Top)
MCOLN2 shRNA #2	CCGGGCATGATATGAAGCAACTGTTCTCGAGAACAGTTGCTTCATATCATGCTTTTTTG(Top)

Table s3 The oligonucleotides sequence for siRNAs

Gene	Sequences (5'-3')
MCOLN2 siRNA1	GCUCGAUUGUGUUCAAUUATT
MCOLN2 siRNA2	GCAUAUAUGAAGCUGUU
mTOR siRNA	AAAGAUCUCAUGGGCUUTT

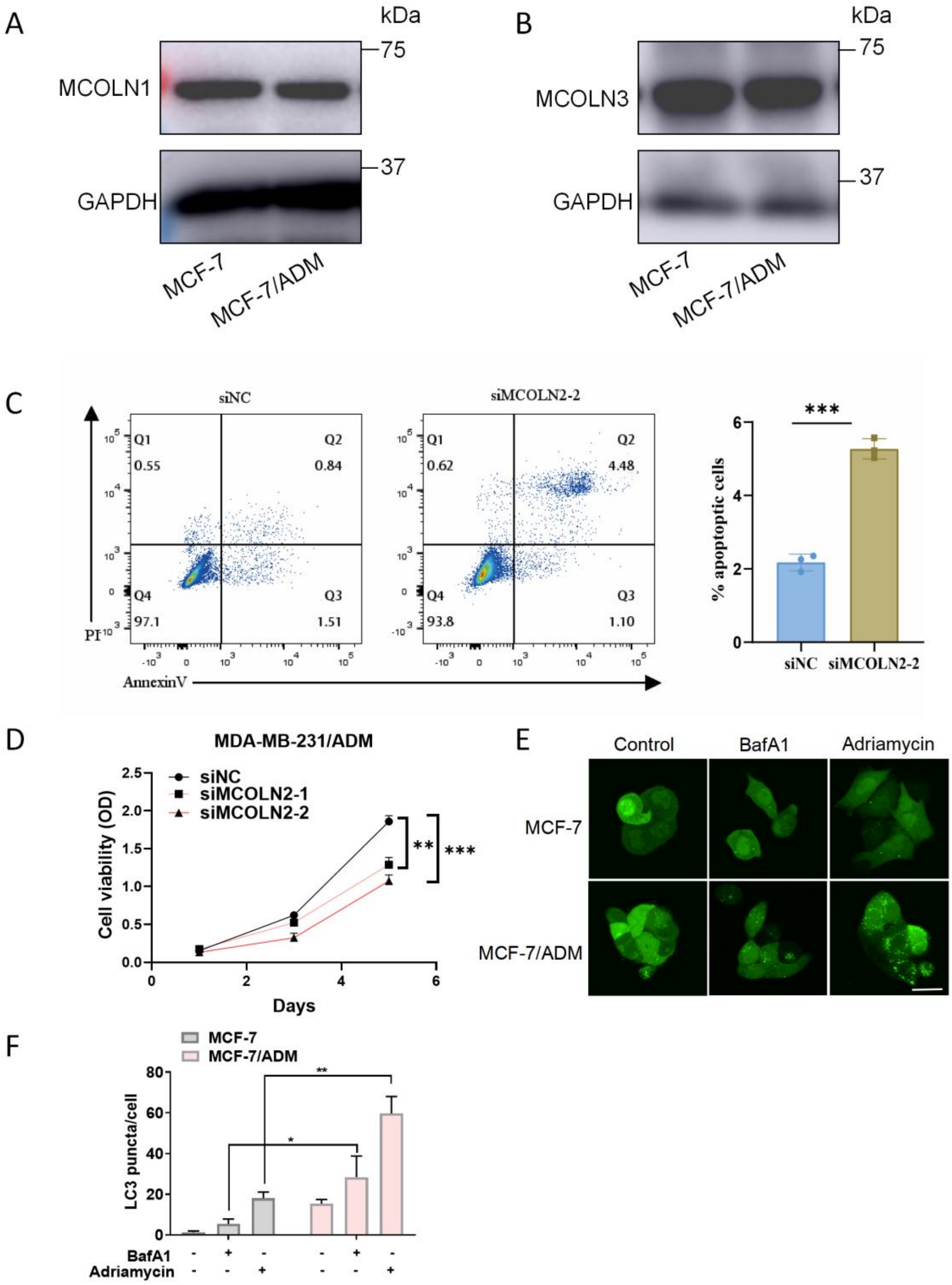

Supplemental Figure 1. (**A-B**) The endogenous expression levels of MCOLN1 (A) and MCOLN3 (B) proteins in Adriamycin resistant (MCF-7/ADM) and their parental cells (MCF-7) were detected by immunoblots. (**C**) Flow cytometry analysis of apoptosis in cells transfected with siMCOLN2-2 or siNC.The left panel shows scatter plots of Annexin V/PI staining, and the right panel presents the percentage of apoptotic cells (***$p < 0.001$). Data represent mean ± SD from three independent experiments. (**D**) The MDA-MB-231/ADM cells were transfected with MCOLN2 siRNAs or siNC. 48 hr post transfection, the cells were incubated with 1 μM Adriamycin for different time durations. The relative cell viability was monitored by CCK8 assay. **(E-F)** The MCF-7/ADM and MCF-7 cells were infected with the Ad-GFP-LC3 virus for 24 hr, then incubated with Adriamycin (2 μM for MCF-7/ADM and

1 μM for MCF-7 cells) for 24 hr or incubated with 100 nM BafA1 for 3 hrs. The LC3 puncta in each cell were measured by Image J. Shown were representative images (scale bar:20 μm) (E) and data summary (n = 20 cells/sample examined over three independent experiments, Mean ± SD) (F).

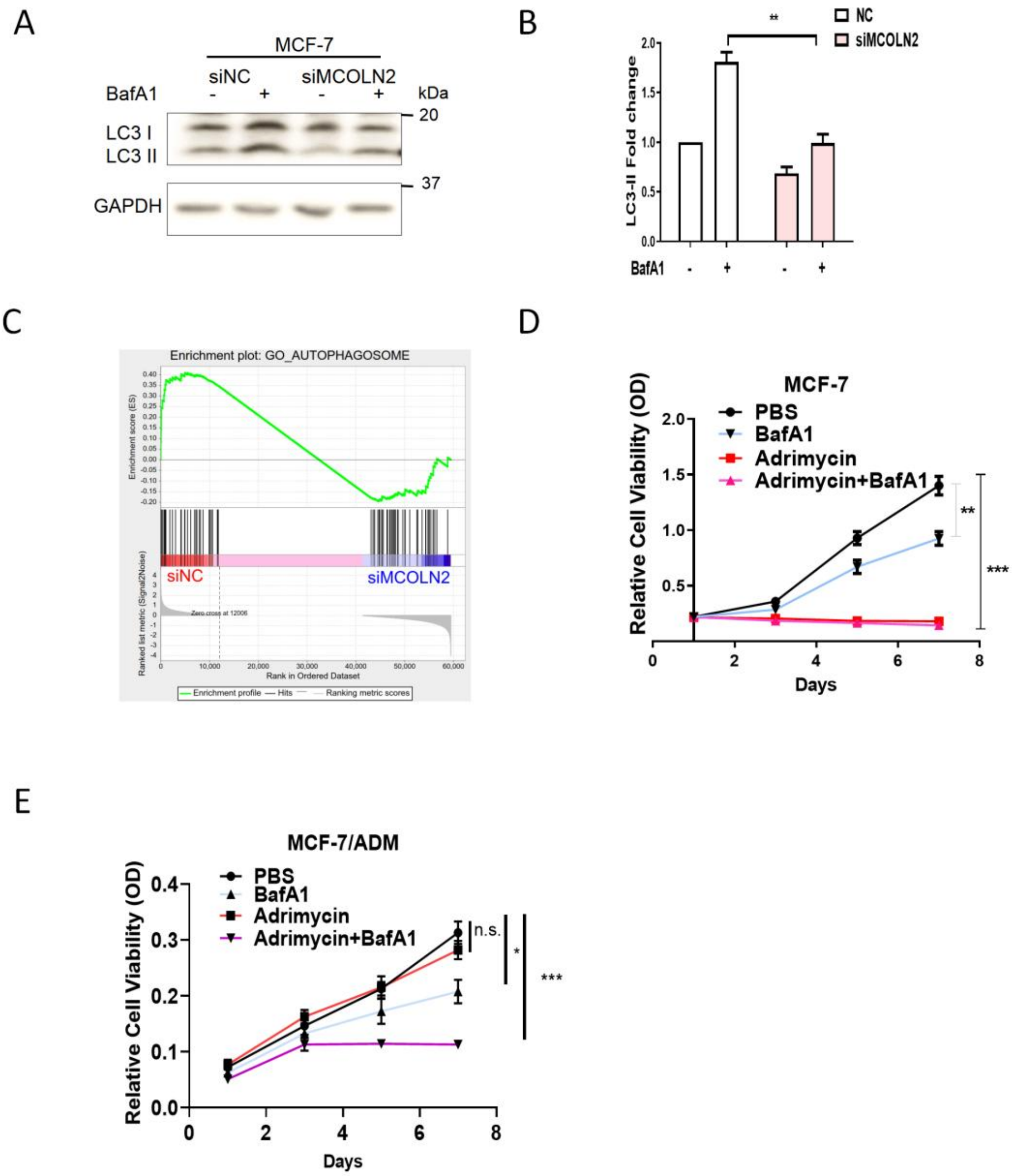

Supplemental Figure 2. (A-B) The MCF-7 cells were transfected with siMCOLN2 or siNC. 48 hr post transfection, the cells were incubated with 100 nM BafA1 for 3 hr. Shown were representative images (A) and data summary normalized to GAPDH (B). Mean ± SD, n = 3. **(C)** GSEA analysis showed that the genes negatively associated with autophagosomes were enriched in siMCOLN2 group compared to the siNC group. **(D-E)** Inhibiting autophagy by 10 nM Baf A1 decreases the Adriamycin resistance in MCF-7/ADM cells**.** MCF-7/ADM and MCF-7 cells were treated with 10 nM Baf A1 and Adriamycin (2 μM for MCF-7/ADM and 0.5 μM for MCF-7 cells) for the indicated time period. Cell viability was monitored by CCK8 assays in 3 -5 independent experiments. Mean ± SD (n = 3-5). **, $P < 0.01$.

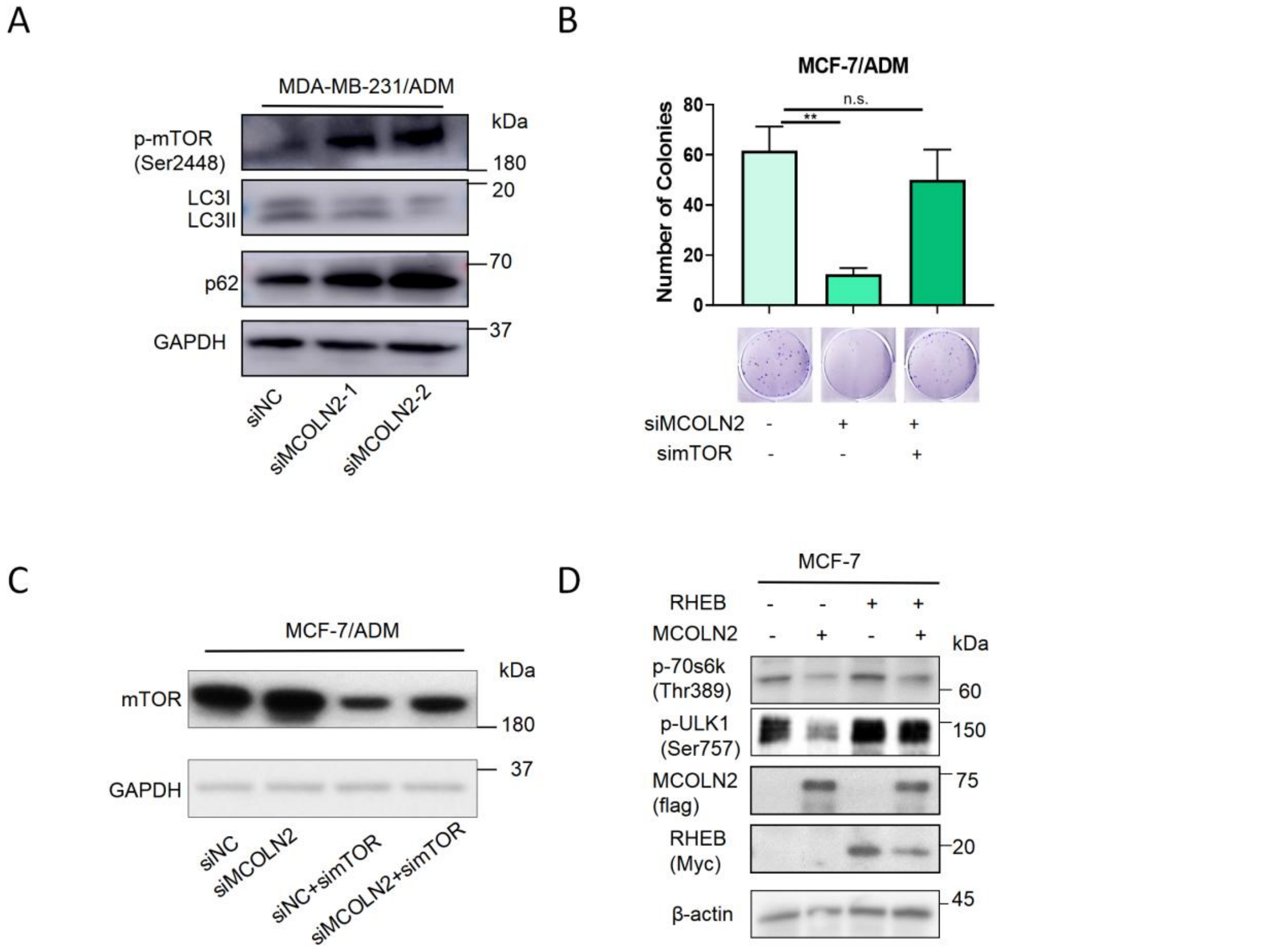

Supplemental Figure 3. (A) Representative immunoblot images showing MDA-MB-231/ADM cells transfected with siMCOLN2-1, siMCOLN2-2, or negative control siNC. 48 hr post transfection, the cells were incubated with 1 μM Adriamycin for 24 hr. **(B)** MCF-7/ADM cells were co-transfected with siMCOLN2 and/or si-mTOR, then incubated with Adriamycin for indicated time periods. The relative cell viability was monitored by colony formation assays. *, P< 0.05; **, P< 0.01.**(C)** Effectiveness of si-mTOR in knocking-down mTOR protein expression in MCF7/ADM cells. **(D)** Shown were representative immunoblots of p-mTORs' substrates in MCF-7 cells transfected with MCOLN2 (+), RHEB(+) or empty vector plasmid (D). n = 3.

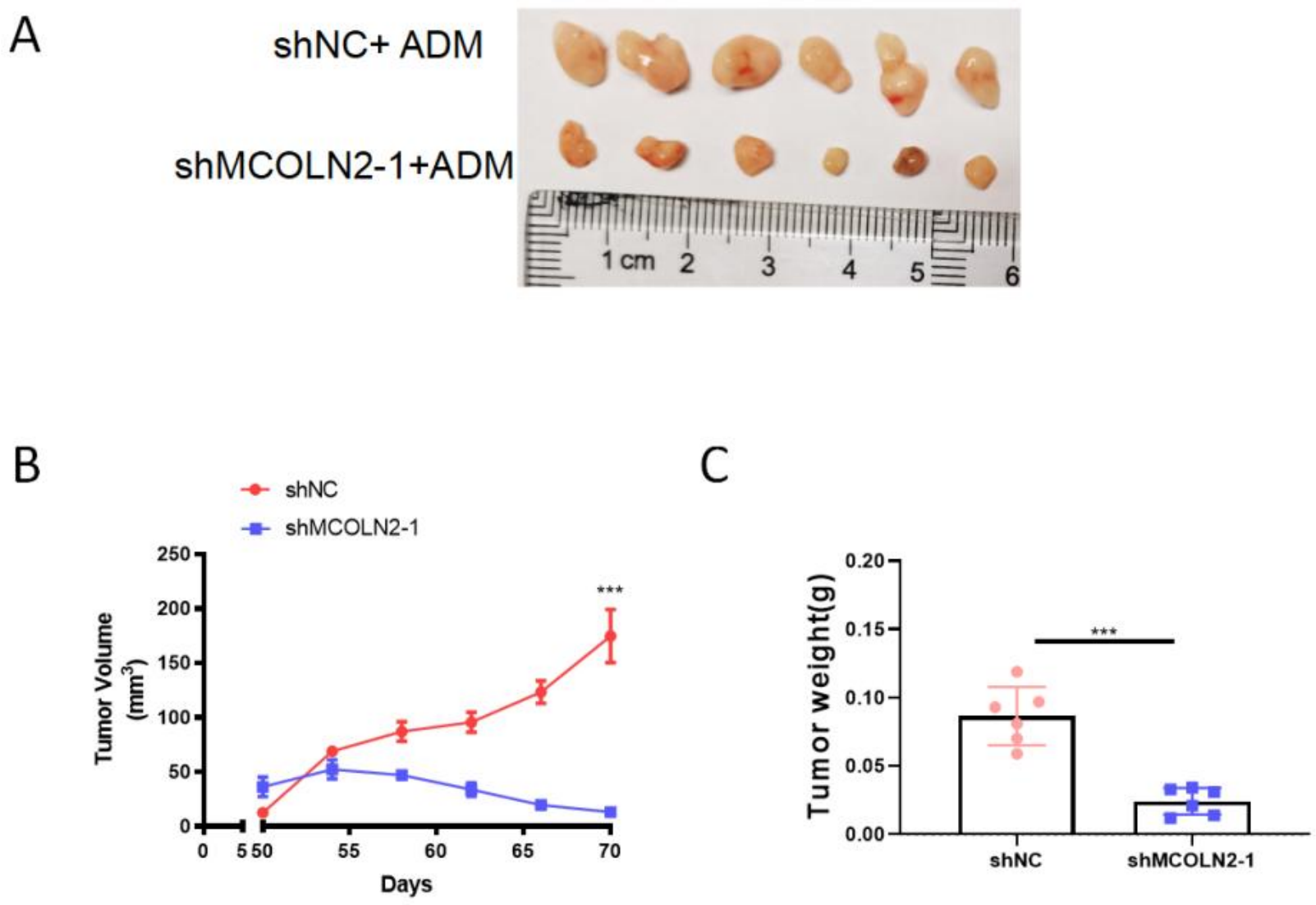

Supplemental Figure 4. MCOLN2 knockdown reverses the adriamycin resistance *in vivo*. **(A)** The effect of Dox-inducible MCOLN2 knockdown by shMCOLN2-1 on the growth of tumor xenografts under adriamycin in NCG mice. Shown were the images of xenograft tumors harvested at the end of the experiment. **(B)** Growth curves of tumor xenografts. **(C)** The weights of tumors at end point are presented as a dot plot. Mean ± S.D (n = 6/group). ***, P< 0.001.
